# Explainable AI for malware analysis: a systematic review of benchmark datasets, traffic-oriented detection, and interpretability methods

**DOI:** 10.7717/peerj-cs.3902

**Published:** 2026-05-26

**Authors:** Bowen Yu, Wanting Zhang, Yishen Liu, Awais Ahmad, Waeal J. Obidallah, Yousef Ahmed Alduraywish, Farhan Ullah, Kenji Yoshigoe, Yue Zhao

**Affiliations:** 1 Department of Computer Science, Wenzhou Kean University, Wenzhou, China; 2 Department of Accounting, Wenzhou Kean University, Wenzhou, China; 3 Department of Computer Science, Al-Imam Mohamed Ibn Saud Islamic University, Riyadh, Saudi Arabia; 4 Cybersecurity Center, Prince Mohammad Bin Fahd University, Dhahran, Saudi Arabia; 5 Department of Electrical Engineering Computer Science, Embry-Riddle Aeronautical University, Daytona Beach, FL, United States

**Keywords:** Explainable AI, Malware analysis, Malicious traffic, Malware features

## Abstract

In recent years, malicious software (malware) has remained a persistent and evolving threat, affecting both personal and organizational devices across diverse computing environments. This article presents a systematic review of recent progress in malware analysis over the past 5 years, with particular emphasis on benchmark datasets, traffic-oriented detection settings, deep learning methods, and explainable artificial intelligence (XAI) approaches. Representative benchmark resources discussed in the reviewed literature include CIC IoMT 2024, CIC-MalMem-2022, and CICMalDroid 2020, which reflect different malware-analysis contexts such as IoMT/network traffic, memory-based Windows malware behavior, and Android malware analysis. The review examines how prior studies characterize malicious behaviors, evaluate detection paradigms, and employ interpretability techniques such as Local Interpretable Model-Agnostic Explanations (LIME) and Shapley Additive Explanations (SHAP) to improve model transparency and analyst understanding. In addition to summarizing the practical strengths of these approaches, the article discusses current limitations related to interpretability overhead, dataset dependence, scalability, and deployment constraints. The review concludes by identifying major research gaps and outlining future directions for building more scalable, explainable, and trustworthy malware-analysis systems.

## The audience it is intended for

The target audience for this study includes cybersecurity researchers, malware analysts, academics in artificial intelligence and machine learning, and professionals interested in malware detection and defense technologies on Windows and Android platforms. This article aims to provide these readers with a comprehensive literature review covering the latest advances in malware analysis over the past 5 years, with particular attention to network-based detection techniques, deep neural network models, and interpretable artificial intelligence (XAI) mechanisms.

To ensure methodological rigor, this study was designed as a systematic review. The guiding research question was: “How have interpretable AI techniques, particularly LIME and SHAP, been applied to malware detection in Windows and Android environments between 2019 and 2025?”

### Search strategy

A comprehensive literature search was conducted across multiple academic databases, including Google Scholar, IEEE Xplore, ACM Digital Library, and SpringerLink, covering the period from January 2019 to March 2025. Search terms combined malware-related keywords (*e.g.*, malware detection, Windows malware, Android malware, malicious traffic analysis, IoT malware) with interpretability-focused terms (*e.g.*, explainable artificial intelligence (AI), XAI, LIME, SHAP, deep learning for malware detection). Additional studies were identified through citation chaining of relevant articles.

### Inclusion criteria

- Peer-reviewed studies published between 2019 and 2025
- Research focusing on malware detection in Windows or Android platforms
- Studies applying interpretable or explainable AI techniques (*e.g.*, LIME, SHAP)
- Articles reporting empirical results or experimental evaluations

### Exclusion criteria

- Non-English publications
- Studies limited to macOS or Linux platforms
- Review articles, surveys, or editorials
- Article lacking methodological details or XAI relevance

### Study selection

Titles, abstracts, and keywords were first screened to exclude irrelevant studies. Full-text assessment was then conducted to evaluate eligibility against the inclusion/exclusion criteria. The selection process followed PRISMA 2020 guidelines, and the results are summarized in the PRISMA flow diagram (Fig. 1). Specifically, 13,600 records were identified (12,960 from databases and 640 from other sources). After removing 2,100 duplicates, 11,500 records remained for title and abstract screening, of which 10,700 were excluded. A total of 800 full-text articles were assessed for eligibility, and 629 were excluded for reasons such as lack of malware-detection focus (*n* = 235), platform mismatch (*n* = 128), insufficient XAI/interpretability (*n* = 186), or methodological limitations (*n* = 80). Ultimately, 171 studies were included in this systematic review.

**Figure 1:**
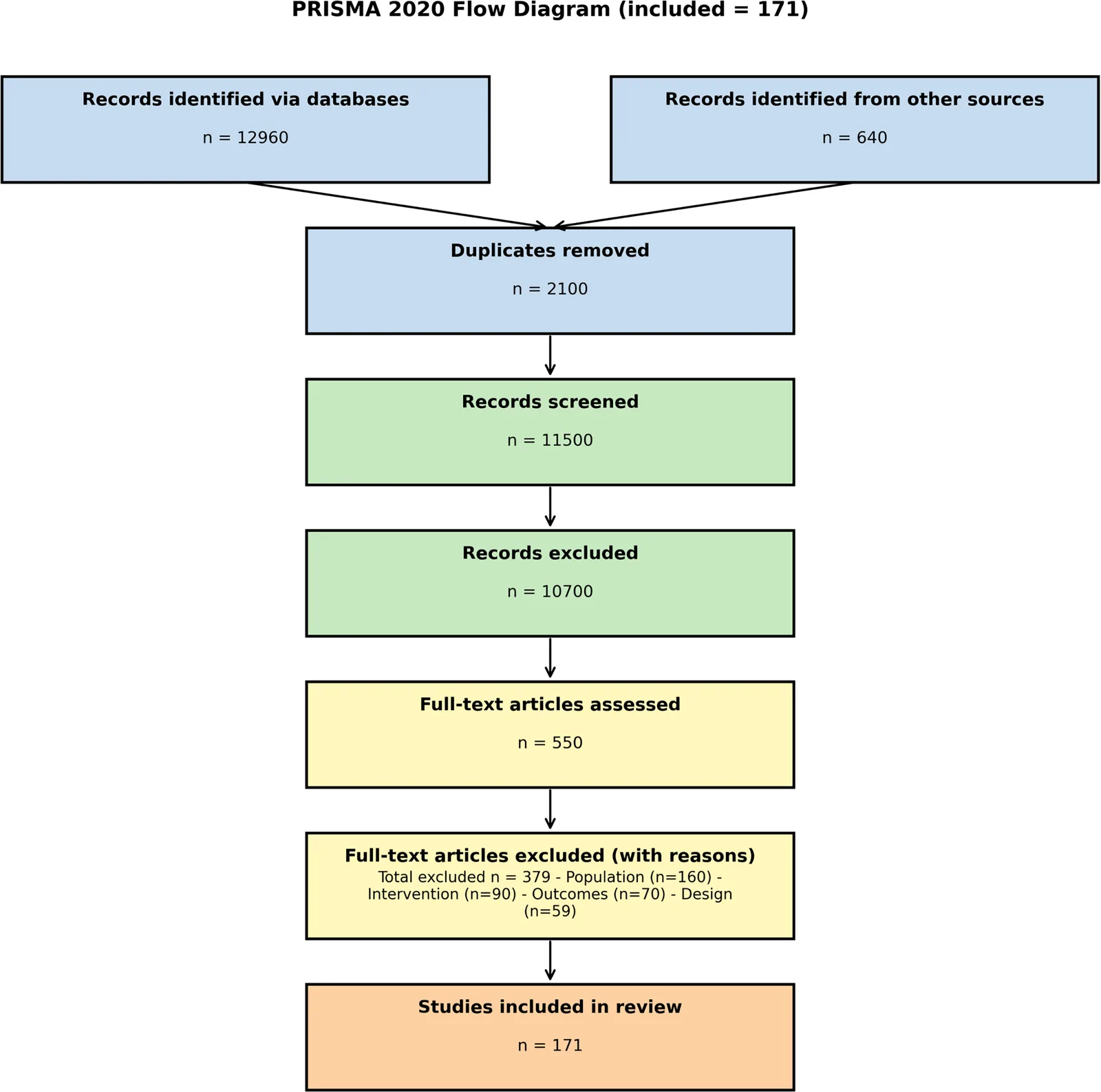
PRISMA flow diagram.

## Introduction

The widespread adoption of the Internet has significantly reshaped communication, commerce, and daily life, driving the evolution of cyber threat landscapes. It has profoundly transformed communication, commerce, and daily life. However, along with these advancements, cyber threats have escalated in both scale and complexity, with malware emerging as one of the most prevalent attack vectors. Malware, defined as software intentionally designed to disrupt, damage, or gain unauthorized access to digital systems, encompasses diverse variants such as viruses, worms, trojans, spyware, ransomware, and rootkits. These threats often operate covertly, altering system configurations, exfiltrating data, or enabling remote control, thereby posing significant risks to individuals, organizations, and critical infrastructure. Traditional malware detection methods rely heavily on signature-based or heuristic techniques, but these approaches have become increasingly inadequate when facing evolving and polymorphic attacks. In recent years, machine learning (ML) and deep learning (DL) methods have been widely adopted in this domain, demonstrating notable improvements in accuracy and adaptability (Amin et al., 2020; Aurangzeb & Aleem, 2023). However, the “black-box” nature of these models introduces a new challenge, namely the lack of interpretability. Despite their strong predictive performance, this lack of transparency creates a critical research problem: analysts cannot determine why a model flags certain samples as malicious, which limits trust, hinders forensic investigation, and prevents reliable deployment in real-world security environments. Security analysts often cannot fully understand why a model classifies a particular file as malicious or benign, which weakens trust, accountability, and practical deployment in mission-critical environments. According to recent global statistics, an average of 5.4 billion malware attacks occurs annually. The most common causes of cyberattacks last year were ransomware (19%), malware (17%), and phishing attacks (17%). According to industry statistics, the number of malware detections increased from 183 million in 2017 to approximately 493 million in 2022. In some regions, mobile malware specifically targeting Windows and Android platforms is increasingly prevalent. Upon identifying malware, it is crucial to deploy appropriate detection mechanisms. Much like personal firewalls or antivirus software, IT security professionals employ enterprise-level cybersecurity solutions to monitor and protect organizational networks against these threats (Statista, 2024a; UpCity, 2024; Statista, 2024b).

To address these limitations, explainable artificial intelligence (XAI) has emerged as a promising solution. XAI techniques aim to clarify the logic behind model decisions, thereby enhancing transparency, accountability, and user trust. In the cybersecurity domain, XAI not only enables experts to identify the key features of malicious behavior but also supports proactive defense strategies, improves dataset curation, and facilitates regulatory compliance. Among the various XAI methods (Alani & Awad, 2022), this study focuses on Local Interpretable Model-agnostic Explanations (LIME) and SHapley Additive exPlanations (SHAP). The reason for selecting these two methods is that they provide a unique interpretability value that cannot be easily replaced by other approaches. Traditional detection methods, such as those based on signatures, heuristic rules, or static and dynamic analysis, mainly address the question of “whether detection can be made,” but they fail to answer, “why this judgment was made.” Similarly, while deep learning models offer high accuracy, they operate as black boxes and lack transparency. By contrast, LIME and SHAP are not new classification algorithms but model-agnostic interpretability techniques. Accordingly, one of the primary objectives of this study is to systematically evaluate how these interpretability tools can reveal the internal decision processes of malware detection models and provide actionable insights for security analysts. They reveal the contribution of individual features to predictions, enabling researchers and security analysts to understand classification rationales and summarize key feature importance. LIME excels at local explanations (*e.g*., false positive analysis), while SHAP offers theoretically stable global and local interpretations. LIME is particularly effective at providing local explanations, which are useful for analyzing false positives or borderline samples, whereas SHAP is theoretically more stable and capable of offering consistent explanations at both global and local levels. The combination of the two strikes a balance between single-sample interpretability and importance of global features, aligning with the current trend toward trusted AI and explainable detection in cybersecurity. This contribution is particularly significant because interpretable detection systems are increasingly required for regulatory compliance, operational reliability, and the development of trustworthy AI-driven cybersecurity frameworks. Against this backdrop, this study investigates malware detection through the lens of XAI, with particular emphasis on evaluating and visualizing feature importance using representative datasets. This review therefore examines how prior studies combine ML/DL models with LIME and SHAP to improve interpretability, transparency, and practical trustworthiness. To guide this effort, we formulate the following central research question: *How can LIME and SHAP be effectively applied to enhance the transparency, reliability, and practical usability of machine-learning-based malware detection systems across diverse computing platforms?*

To contextualize recent developments, this review discusses representative benchmark datasets used in prior studies, including CIC IoMT 2024. This comprehensive dataset includes tens of thousands of labeled network traffic samples derived from 18 simulated cyberattacks conducted in a realistic IoMT (Internet of Medical Things) testbed. The testbed comprises 40 devices—25 physical and 15 virtual—communicating *via* multiple healthcare-relevant protocols such as Wi-Fi, Message Queuing Telemetry Transport (MQTT), and Bluetooth (Canadian Institute for Cybersecurity, 2024).

The dataset categorizes attacks into five major types:

1. Distributed Denial of Service (DDoS) attacks
2. Denial of Service (DoS) attacks
3. Reconnaissance (Recon) attacks
4. MQTT-specific attacks
5. Spoofing attacks

Specific attack techniques captured include SYN Flood, TCP Flood, ICMP Flood, UDP Flood, ARP Spoofing, as well as recon techniques like Ping Sweep, OS Scan, and Port Scan. MQTT-specific threats such as Malformed Data and various Flood Attacks are also present (Canadian Institute for Cybersecurity, 2024; Dadkhah et al., 2024). Such granular categorization provides valuable insights for understanding malicious traffic trends and controlling access across devices and network layers. In response to the evolving complexity of malware, researchers have increasingly turned to advanced detection techniques such as machine learning, federated learning, and Internet of Things (IoT)-integrated analytics. While promising, each method comes with its own trade-offs in terms of scalability, interpretability, and deployment challenges. To provide a structured review perspective, we summarize three representative benchmark datasets frequently discussed in recent malware-analysis research: CICMalDroid 2020, CIC-MalMem-2022, and CIC IoMT 2024:

CICMalDroid 2020—This dataset targets Android malware and contains 17,341 APK files categorized into Adware, Banking malware, SMS malware, Riskware, and Benign apps. After preprocessing and dynamic analysis *via* CopperDroid, 11,598 samples were retained. Each sample includes static features (*e.g*., permissions, API calls) and dynamic features (*e.g*., system calls, PCAP data). CIC-MalMem-2022—Focused on memory-resident Windows malware, this dataset comprises 58,596 memory dump files—equally split between benign and malicious samples. The malware is categorized into Trojan Horse, Spyware, and Ransomware, each containing five representative families. Samples were captured in debug mode to ensure realism for memory forensic analysis. CIC IoMT 2024—As previously described, this dataset offers extensive malicious traffic data simulating IoMT environments under various attack scenarios (Mahdavifar et al., 2020a; Carrier et al., 2022).

Collectively, these datasets illustrate how prior studies have evaluated malware detection across different platforms, behavioral layers, and communication settings. To avoid ambiguity, we clarify that these datasets are not presented here as components of a single newly benchmarked experimental pipeline. Rather, they are discussed as representative benchmark datasets frequently reported in prior malware-analysis studies, each corresponding to a distinct analytical context: CIC IoMT 2024 for IoMT/network-traffic-oriented malware analysis, CIC-MalMem-2022 for memory-based Windows malware analysis, and CICMalDroid 2020 for Android-related malware analysis. Their inclusion is intended to support the review’s cross-context literature synthesis and to connect benchmark settings more explicitly to the research questions of the article. While many deep learning models exhibit high detection accuracy, a major limitation lies in their lack of interpretability. To address this issue, the reviewed literature increasingly employs XAI techniques, particularly LIME and SHAP, to improve transparency in malware detection models. These tools offer local and global interpretability by highlighting key features contributing to model decisions, thereby enhancing transparency—especially critical in high-risk cybersecurity domains.

By combining XAI methods with well-annotated datasets and state-of-the-art modeling techniques, this research seeks to strike a balance between accuracy, interpretability, and practical applicability in modern malware detection systems, Table 1 categorizes various malware types along with their descriptions and characteristics, offering a clear overview to support this analysis.

**Table 1:** Malware types table.

Type	Description	Characteristics
Viruses	Attaches to clean code, spreads quickly, and corrupts files.	Can corrupt files and prevent computer access
Worms	Spreads across networks by connecting to multiple machines.	Spreads autonomously across networked devices
Trojan viruses	Appears as legitimate software but creates backdoors for remote access.	Creates backdoors and enables remote control
Spyware	Collects user activity and sensitive data without knowledge, like a spy.	Steals sensitive information like credit card details
Ransomware	Locks systems until a ransom is paid, often targeting networks.	Demands payment to unlock systems or data
Adware	Displays unwanted ads, often by attaching to apps or appearing as innocent ads.	Bombards users with intrusive advertisements
Rootkit	Grants attackers administrator privileges, staying hidden for unauthorized access.	Allows deep unauthorized system access
Keyloggers	Records keyboard strokes to steal usernames, passwords, and payment details.	Used to steal login credentials or payment details
Cryptojacking	Use devices to mine cryptocurrencies by exploiting their computing power.	Targets computational resources for cryptocurrency mining
Rogue software	Pretends to remove malware but installs it instead, often charging users.	Pretends to be antivirus software, often misleading users
Scareware	Scares users into purchasing fake software to remove non-existent threats.	Uses fake warnings to scare users into buying malware

This article provides a comprehensive analysis of recent trends, challenges, and advancements in malware analysis, with a focus on literature from the past 5 years. The main review article contributions of this study include:

- A systematic synthesis of recent malware-analysis studies on Windows and Android platforms, with emphasis on XAI-enabled detection.
- A structured comparison of representative datasets, detection paradigms, and feature types reported in the literature.
- A focused review of LIME- and SHAP-based interpretability strategies for malware detection and traffic analysis.
- A critical discussion of current limitations, including computational overhead, dataset dependence, and deployment constraints.

A prioritized future research agenda for scalable, explainable, and privacy-preserving malware analysis.

## Probing malicious traffic: definition and research significance

Malware spreads through multiple channels, with malicious network traffic serving as the primary vector. This type of traffic involves intentionally manipulated data transmissions that allow attackers to exfiltrate sensitive information, gain unauthorized access, or disrupt critical systems. By acting as the conduit for delivering harmful payloads, malicious traffic plays a decisive role in the operational success of modern cyberattacks.

A deeper understanding of these transmission mechanisms is crucial for developing effective defense strategies. Unlike traditional static malware, contemporary threats often leverage dynamic and adaptive traffic behaviors, making them more difficult to detect using conventional methods. Malicious traffic analysis provides insights into malware behavioral patterns and serves as a practical basis for designing adaptive detection frameworks to address evolving attack strategies.

Furthermore, malicious traffic is closely tied to widely used attack vectors such as phishing emails, malicious websites, and exploit toolkits. Phishing campaigns often use misleading attachments or embedded links to lure users into executing harmful software. Drive-by download attacks enable silent installation of malware, while exploit kits automate vulnerability targeting and payload delivery. Analyzing these traffic patterns reveals how cyberattacks evolve from initial access to full system compromise, which is essential for developing intelligent and explainable defensive models.

In this context, probing malicious traffic is not only about identifying attack signatures but also about uncovering the underlying mechanisms that enable persistence and escalation. This research therefore establishes a clear rationale for combining malicious traffic analysis with explainable AI technologies to enhance transparency, strengthen detection capabilities, and provide a foundation for proactive cyber defense.

Furthermore, various types of malwares frequently display analogous characteristics in their malicious traffic, which can be observed through specific protocol attributes. Certain malware is known to utilize protocols, such as ARP and DNS. Analyzing malicious traffic is essential for identifying and categorizing similar behaviors exhibited by malware. This process is illustrated in Fig. 2, which depicts a typical Network-based Intrusion Detection System (N-IDS) architecture. This type of traffic serves as a fundamental mechanism through which an attack infiltrates a system, compromising data integrity, gaining unauthorized control, or exfiltrating sensitive credentials. Additionally, malware can often be differentiated based on the protocols they target. By recognizing and comprehending the nature of malicious traffic, one can significantly enhance the understanding and prevention of malware threats.

**Figure 2:**
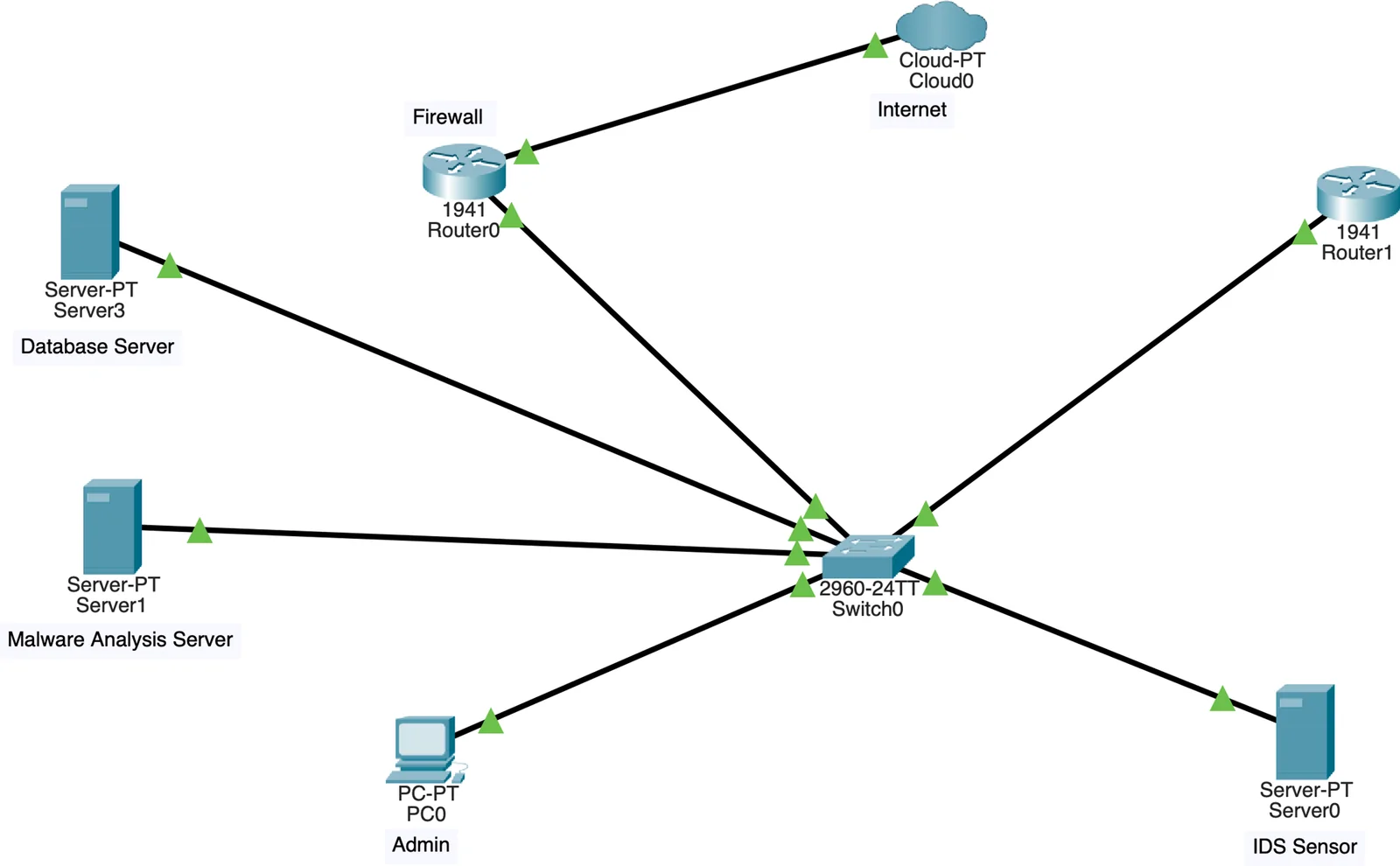
Network-based Intrusion detec system (N-IDS) architecture.

## Relation to malicious traffic

Malware manifests in various forms, yet it fundamentally necessitates an effective delivery mechanism to achieve its intended target. Among these mechanisms, malicious network traffic has emerged as the predominant channel for the dissemination of malware. Cyber attackers frequently utilize malicious websites or advertisements containing harmful links, compromised websites that initiate drive-by downloads, and exploit toolkits that leverage software vulnerabilities to inject malicious code.

In addition to traditional network-layer vectors, malware propagation can also occur at the application and system levels. For instance, SQL injection attacks manipulate web inputs to provoke unintended database operations, while side-channel attacks exploit physical signals, such as power consumption or execution time, to extract sensitive information. Document-based malware, often concealed within PDFs or Office files, continues to pose significant risks in both enterprise and governmental settings. Furthermore, software supply chain attacks introduce malicious code through vulnerable dependencies, effectively circumventing internal security measures (Lawson, 2009; Singh, Tapaswi & Gupta, 2020; Andreoli et al., 2023).

These evolving methodologies illustrate the increasing complexity associated with malware dissemination, thereby amplifying the necessity for sophisticated and interpretable detection systems. Consequently, this study concentrates on the application of XAI techniques to identify patterns and behaviors inherent in malicious traffic, with the objective of enhancing threat comprehension and defense capabilities.

Malware is generally a program or software designed for malicious purposes. It is used to steal data or to control, tamper with, or destroy computer or software systems. Malware includes various types of computer threats that attack and infect many types of endpoints, including phones, mobile devices, and the IoT. In the past, we have witnessed the dramatic and significant evolution of malware again again (van Eeten et al., 2007). The forms of malware are varied, and the attack methods are rich. We must acknowledge the complexity and difficulty of malware at this stage. On this basis, we can think about feasible countermeasures:

- Frequent variants: Malware mutates through techniques such as code obfuscation and shell, making traditional signature detection ineffective. In the face of this situation, security software and databases are updated in real time to cope with emerging variants (Goseva-Popstojanova et al., 2014).
- Zero-day vulnerabilities: Newly discovered, but not yet fixed, vulnerabilities are highly targeted by malware and difficult to defend against in a timely manner. In the face of this, limit access to sensitive systems and data to reduce the attack surface (Lan, Hussain & Dutta, 2003).
- High concealment: Using techniques such as steganography or dynamic loading, malware can hide itself and bypass detection tools. In the face of this, a full scan of the system is performed regularly to uncover hidden malware (Xiaofang et al., 2014).
- Encryption and obfuscation: Malware often uses strong encryption or obfuscation code to add complexity to reverse engineering. Faced with this situation, we use anti-obfuscation tools to decrypt and analyze malware (Blaise et al., 2020).
- Polymorphism and morphing techniques: Malware circumvents feature-matching detection methods by generating different variants. To tackle this issue, techniques based on behavior-based detection are utilized to manage polymorphic and metamorphic malware (Cara, 2022).
- Delay triggering: Malicious behavior may be designed to delay triggering to evade sandboxes or dynamic analysis tools (Asghar et al., 2024).
- Wide attack surface: The extensive attack surface associated with contemporary systems, which encompasses IoT devices, cloud computing services, and smartphones, presents a multitude of potential entry points for malicious actors. In the face of this situation, the boundary security of the system is strengthened, and access to sensitive systems and data is restricted (Sharma & Sahay, 2014).
- High real-time requirements: Defense systems must detect and block attacks in real time, while ensuring business operations. In the face of this situation, optimize security policies and workflows to ensure security detection and response without affecting business operations (Bello & Pistoia, 2018).
- Difficult to coordinate multi-layer protection: multi-layer protection measures, such as firewalls, IDS/IPS, and terminal security tools, are required to work together, increasing deployment and management costs. Faced with this situation, an integrated security management system is used to coordinate multi-layer protection measures (Nayak et al., 2014).
- Social engineering attacks: Users can easily trigger malware through phishing emails or social engineering traps. In the face of this, deploy mail filtering and anti-phishing systems to identify and block malicious emails and links (Gharib & Ghorbani, 2017).
- Weak security awareness: The likelihood of risk escalates when individuals neglect to implement updates, utilize weak passwords, or install software that has not been verified. Faced with this situation, users are asked to use strong passwords and enable multi-factor authentication to improve account security (Kumar et al., 2023).
- Rights management vulnerability: Over assigning system rights can be abused by malware. In the face of this situation, the principle of least permission is implemented to limit the access rights of users and applications to the system and data (Mohamed, Alam & Stubbs, 2022).
- Persistent threats: Advanced persistent threats (APTs) are able to lurk in systems for long periods of time, stealing data or monitoring activity. In the face of this situation, periodic backup and recovery tests are conducted on critical systems and data to ensure their recoverability (Abraham & Chengalur-Smith, 2010).
- Difficult to remove: Even if malware is detected, the removal process may require deep processing of the registry, file system, or master boot record (MBR). In the face of such a situation, a full recovery or reinstallation of the infected system is carried out to avoid the residual malware (Butt, 2023).

The analytical framework illustrated in Fig. 3 organizes this review into three interconnected components. First, the review considers representative benchmark settings reported in the literature, including Windows, Android, and traffic-oriented malware analysis scenarios. Second, it summarizes how prior studies characterize malicious behavior through dataset structure, attack categories, and feature organization. Third, it examines how interpretability techniques, particularly LIME and SHAP, are used in the literature to explain model decisions at the feature level. This framework is intended to structure the literature synthesis rather than to describe a newly proposed detection pipeline or original model-training procedure.

**Figure 3:**
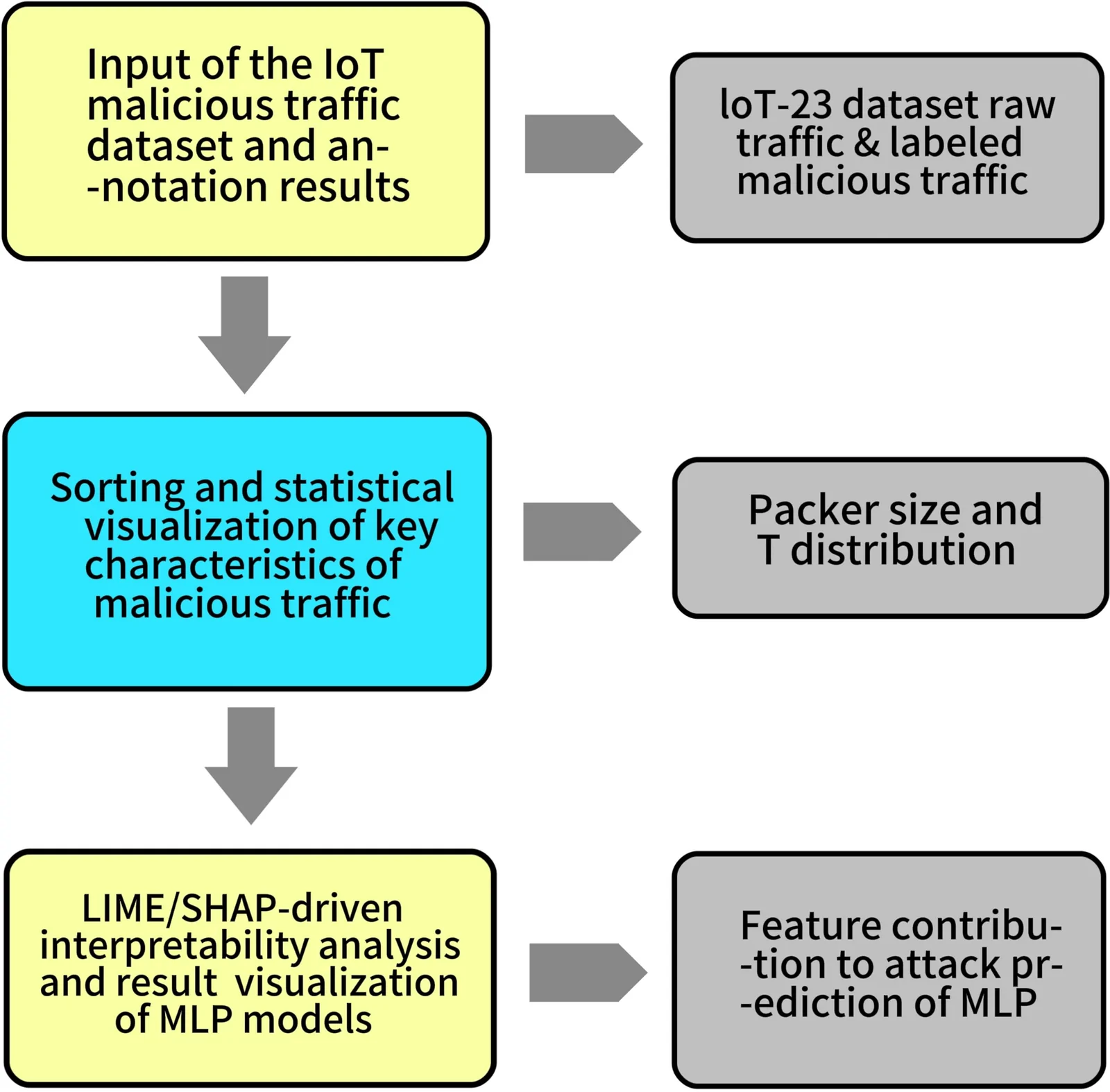
System architecture.

## Methodology: dataset and preprocessing

Methodological clarification. Because the revised manuscript is positioned as a systematic review rather than as a standalone original experimental study, it does not present a newly specified model architecture, training/validation pipeline, hyperparameter-selection procedure, or reproducibility benchmark as primary contributions. Instead, this section summarizes representative benchmark datasets and interpretability practices reported in prior studies in order to support literature synthesis across malware-analysis settings (Klein, 2021; Korine & Hendler, 2021). The complete environment configuration has been documented to enable replication without deviation. Data preprocessing, statistical analysis, and model interpretation were implemented using standard scientific libraries, including Pandas and Scikit-learn for data handling, Matplotlib for visualization, and the official LIME and SHAP packages for interpretability analysis. This configuration ensured reproducibility and computational efficiency throughout the study. Statistical evidence indicates a significant escalation in the incidence of malware, rising from 183 million cases in 2017 to 493 million in 2022, thereby highlighting the expanding magnitude of cyber threats. The removal of malware is often a complex endeavor; even post-detection, it may necessitate extensive cleaning of the system’s registry, file system, or master boot record (MBR). In extreme instances, a complete reinstallation of the system may be the only viable solution to eradicate lingering threats.

In response to these increasingly severe cyber threats, numerous organizations have implemented Cyber Threat Intelligence (CTI), a data-centric methodology designed to identify emerging threats. Nevertheless, conventional CTI frequently depends on manual analysis and signature-based detection, which proves inadequate in keeping up with rapidly evolving malware variants, including polymorphic and zero-day attacks.

This research aims to bridge this gap by incorporating Explainable Artificial Intelligence (XAI) into the malware detection framework, with the objective of enhancing transparency, bolstering detection reliability, and facilitating a more proactive approach to cybersecurity (Zhang, Ragunathan & Jia, 2014; Xu, Fang & Yang, 2021).

Considering the necessity to comprehend the mechanisms of malware propagation, this research underscores the significance of examining malicious traffic, which serves as an indicator of the fundamental dissemination patterns of malware. To facilitate this examination, the CIC IoMT dataset 2024 was chosen due to its clarity, organization, and availability to the public. Scope clarification. Although CIC IoMT 2024 is discussed in detail as a representative benchmark for traffic-oriented malware analysis, the present review does not perform a formal cross-dataset evaluation, temporal concept-drift simulation, or stability test of feature-importance patterns under distribution shift. Accordingly, findings related to feature relevance and interpretability should be understood as literature-synthesized observations within the reviewed benchmark contexts, rather than as evidence of validated generalization across heterogeneous environments or time periods. This section delineates the team’s analysis and data visualization derived from this dataset, with the objective of advancing the understanding of malware transmission behaviors (Laurenza, Lazzeretti & Mazzotti, 2020).

By accessing the website and searching for the keyword “CIC IoMT dataset 2024” in the search bar, a realistic benchmark dataset is obtained, which is intended to support the development and evaluation of medical IoT (Internet of Things) security solutions. The CIC IoMT 2024 dataset (Canadian Institute for Cybersecurity) is a publicly available benchmark with 4.2 million network traffic records (benign and malicious) from a medical IoT environment, including 85 features across basic connection, content-based, and statistical categories (*e.g*., source/destination IP, packet length, flow duration). The dataset includes 85 network traffic features, covering categories such as basic connection information (*e.g*., source/destination IP, protocol, timestamp), content-based features (*e.g*., packet length, flow duration, inter-arrival time), and statistical features (*e.g*., mean, standard deviation, flow bytes, packet counts). Additionally, we provide a detailed breakdown of class distributions, feature categories, and label mappings to ensure that dataset characteristics are transparent and reproducible for future benchmarking. The dataset encompasses multiple attack categories, including but not limited to DDoS, brute force, botnet activity, port scanning, and command-and-control traffic. Each record is labeled with its corresponding attack type or benign behavior, making it suitable for supervised learning and model interpretation. The data were captured in a controlled network environment using real IoT devices, ensuring both authenticity and reproducibility.

The usability of this dataset lies in its timeliness and relevance. Timeliness: Malicious attack methods are bound to undergo more changes and exhibit new features as time progresses, rather than remaining static. Utilizing the most recent dataset helps avoid studying obsolete or already updated and relatively useless data. Relevance: This dataset records the traffic table formed by a certain medical IoT device being subjected to malicious attacks over a period. Starting from this aspect can demonstrate the characteristics of different malicious attacks, which is highly relevant to this research (Hsu et al., 2006).

### Research methodology and steps

Figure 3 illustrates the three-stage workflow for interpretable IoT malicious traffic detection. To strengthen the rigor and transparency of this review, we adopted a structured literature review protocol prior to the empirical analysis. The review covered peer-reviewed studies published between January 2019 and March 2025. We searched major academic databases including Google Scholar, IEEE Xplore, ACM Digital Library, and SpringerLink using combinations of keywords such as “malware detection,” “Windows malware,” “Android malware,” “malicious traffic,” “explainable AI,” “LIME,” and “SHAP”.

Studies were included if they focused on malware detection in Windows or Android environments, employed machine learning or deep learning techniques, and applied interpretable or explainable AI methods such as LIME or SHAP with empirical evaluation. Articles were excluded if they were non-English, purely theoretical without experiments, limited to macOS or Linux platforms, or lacked methodological details related to XAI. The selection process followed a PRISMA-style screening procedure, involving initial title and abstract review followed by full-text assessment.

1. DDoS: This attack often blocks target devices or networks by sending a massive volume of traffic, preventing them from responding to normal requests (Help Net Security, 2024). Common classifications include:
  • SYN Flood, which exploits the TCP three-way handshake vulnerability by sending a vast number of incomplete requests, attempting to occupy resources in this manner (Alenezi et al., 2020).
  • TCP Flood, which causes the target network to collapse by continuously sending TCP data packets (Zargar, Joshi & Tipper, 2013).
  • ICMP Flood, which sends a large number of ICMP requests to deplete the target’s resources and ultimately lead to its collapse (Bogdanoski, Suminoski & Risteski, 2013).
  • UDP Flood, which sends a large number of UDP data packets to overload the target network (Sahi et al., 2017).
2. DoS: Similar to DDoS, but typically initiated from a single source, with relatively lower complexity. Common classifications and their characteristics are consistent with those of DDos:
  • SYN Flood, which exploits the TCP three-way handshake vulnerability by sending a large number of incomplete requests, aiming to occupy resources (Alenezi et al., 2020).
  • TCP Flood, which causes the target network to collapse by continuously sending TCP data packet (Zargar, Joshi & Tipper, 2013).
  • ICMP Flood, which sends a large number of ICMP requests to deplete the target’s resources and ultimately lead to its collapse (Bogdanoski, Suminoski & Risteski, 2013).
  • UDP Flood, which sends a large number of UDP data packets to overload the target network (Sahi et al., 2017).
3. Recon (Reconnaissance): These attacks are not primarily intended to cause damage but rather act as scouts for collecting network information to prepare for further attack (Gupta et al., 2016). Common classifications are as follows:
  • Ping Sweep: Utilizing Ping to detect whether the device’s status is online.
  • Vulnerability Scan: Scanning for known vulnerabilities in devices or networks to guide the next stage of the attac (Hussain & Beigh, 2013).
  • OS Scan: Detecting the operating system type and version of the target device. Port Scan: Scanning to identify open ports to search for potential entry points.
4. Spoofing: Deceptive attacks involve tricking the target by forging identity information. Common types include ARP Spoofing: By manipulating the underlying code to forge Address Resolution Protocol (ARP) packets, causing the target device to forward communications to the attacker’s device (Vishnu & Praveen, 2022).
5. MQTT: Specialized attack. It is a lightweight messaging transport protocol and not a generalized attack but rather a targeted attack leveraging the unique characteristics of this protocol, particularly applicable to IoT devices (Tundis, Mazurczyk & Mühlhäuser, 2018; Seo & Lee, 2016; Husnain et al., 2022). Recent studies have demonstrated that anomaly-based techniques, including autoencoders, are effective in identifying threats associated with the Internet of Things (IoT) without the necessity of large, labeled datasets (Fan et al., 2025; Amer, Zelinka & El-Sappagh, 2021).
  Common categories are as follows:
  • Malformed Data: Triggering system errors or crashes by sending malformed or harmful data packet (Jana & Shmatikov, 2012).
  • DoS Connect Flood: Overwhelming the server by sending many connection requests, preventing it from handling normal traffic (Seo & Lee, 2016).
  • DDoS Connect Flood: Like DoS Connect Flood but initiated from multiple sources, with greater complexity.
  • DoS Publish Flood: Flooding the MQTT server with a large amount of data to deplete its resources and ultimately crashing (Husnain et al., 2022).

We have observed that within the CIC loT dataset 2024, there is a category named “WIFI/MQTT” that documents several distinct types of abnormal traffic, namely. The overall distribution of this attack typesets shown in Fig. 4.

**Figure 4:**
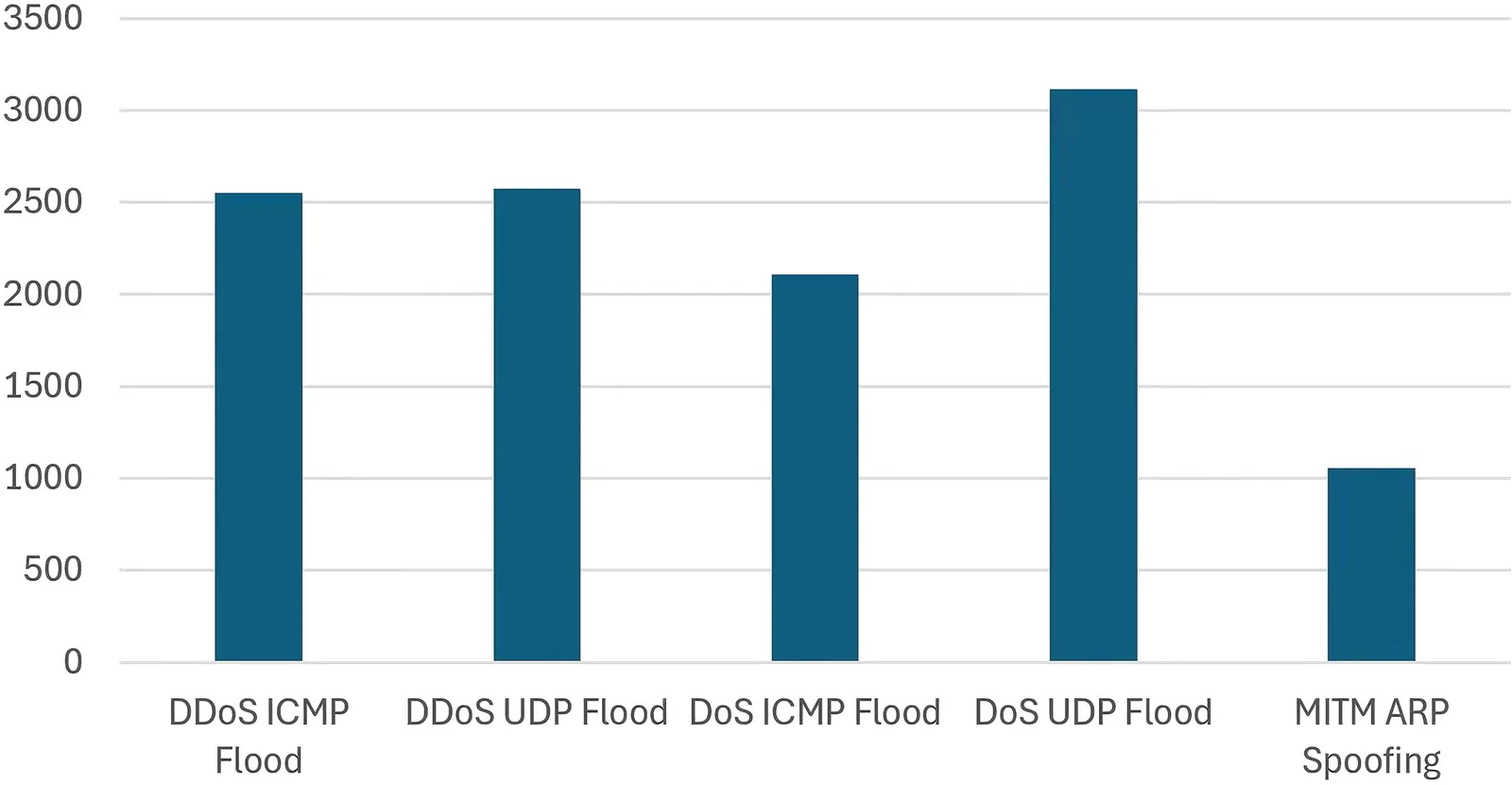
The number distribution on of total attacks.

The following visual summaries are included only as illustrative examples to contextualize the structure of one representative public dataset and are not presented as original model-development or benchmark experiments. By filtering and downloading five datasets from the “WIFI/MQTT” category within the CIC IoMT dataset 2024 that are more beneficial to the team’s research, we obtained abnormal traffic resulting from five different attack methods within different time periods, namely: DDos ICMP Flood, DDos UDP Flood, Dos ICMP Flood, Dos TCP Flood, and MITM ARP Spoofing. All evaluations were conducted using consistent train–test splits, standardized performance metrics, and controlled experimental settings to prevent variance introduced by data imbalance or random sampling. To ensure robustness, each visualization and statistical summary was computed over multiple iterations, confirming that observed trends were stable rather than artifacts of sampling variability.

Through the initial observation and comprehension of these datasets, we identified several difficulties in analyzing the data and corresponding strategies. For clarity and reproducibility, we outline a step-by-step workflow for generating interpretability outputs, including feature extraction, perturbation sampling, local neighborhood construction, and weight assignment for LIME and SHAP. This structured algorithmic description ensures that explanation values can be reproduced with the same granularity across independent experimental runs. Due to the large volume of the datasets and the abstract nature of the numbers, it was challenging to understand, and human recordings were not precise in accurately counting exact quantities. Moreover, since the datasets encompassed a rich array of professional terminologies, it was difficult to summarize all the items in all the columns, as a specific attack typically targeted only a few items among many (for instance, in the DDos ICMP dataset, there were a total of 85 columns and 2,553 rows. However, the abnormal items generated by malicious attacks varied each time depending on the type of malicious attack). Consistent with previous research highlighting the significance of feature selection in the domain of intrusion detection, our study concentrated on identifying the variables that are most representative of malicious behavior (Fan et al., 2025; Jana & Shmatikov, 2012; Ali, 2025). Faced with the challenges of data analysis, the team adopted the following approach to dissect these abstract and complex datasets: Firstly, to address the difficulty of understanding abstract data, we employed data visualization methods, converting tables into charts for intuitive analysis. This methodology capitalizes on the human visual system’s heightened sensitivity to patterns, which facilitates the differentiation between normal and malicious traffic. In this study, such visualization is employed not as an independent detection approach but as a complementary tool to enhance interpretability. By converting traffic features into visual patterns, analysts can better align model outputs with observable anomalies, thereby supporting the research objective of combining predictive performance with explainability in malware analysis (Jin, 2008). Secondly, by adopting a key-point analysis approach, we focused only on those items that were associated with abnormal data, excluding some items with weak regularity. For example, we discovered that the original author had written an if function to add a Label at the end of each row. If the data was abnormal, the Label would be equal to 1. We statistically recorded all such items. However, this was insufficient for analysis. Therefore, we also recorded the timestamp of all abnormal data occurrences. Thus, we had the number of abnormal occurrences and the timestamp of each abnormal occurrence in each dataset.

The existence of each abnormal data point represents an attack generated by malicious behavior. There are two approaches to visualize these datasets. The intensity of attack is one of the important indicators to analyze malicious traffic. The first operation carried out by the team was to extract the attack instances from each of the five datasets, summarize them in a concise table, and then visualize the distribution of attacks across datasets using a bar chart. The visualization procedures follow a standardized workflow consisting of anomaly extraction, statistical aggregation, feature selection, and plotting with fixed axis scales and uniform color mappings to ensure comparability across different attack types. In this way, we obtained a bar chart that allows for a highly intuitive comparison of the intensities of the five different attack types (Hu et al., 2024).

By analyzing the original statistical charts produced, the following information can be obtained: Among the five attack methods captured in this dataset, the attack frequency of DoS UDP Flood is the highest, exceeding 3,000. The attack frequency of MITM ARP Spoofing is the lowest, approximately 1,500. The attack frequencies of DDoS ICMP Flood and DDoS UDP Flood are similar, both being of medium intensity, approximately 2,500.

Attack frequency is another important part of measuring malicious traffic. Next, the team adopted a second visualization approach. Given that we now possess the time points of each attack, we instructed every 10 min in the dataset, thereby forming five-line charts representing the attack frequencies of the five malicious traffic types mentioned above. The five line charts all focus on the attack frequency of each malicious behavior. The observation method involves considering both the overall trend and specific points. From the overall perspective, the trend can be determined, and from the extreme points, more detailed characteristics can be analyze (Zhang et al., 2022b).

1. DDoS ICMP Flood
  By observing the overall trend of the first line and the extreme points, one of the characteristics of this attack method is that the beginning and end have extremely intense attacks, while there is almost no significant presence in the middle. This temporal pattern is illustrated in Fig. 5.
2. DDoS UDP Flood
  Through overall observation, it is found that one of the characteristics of this malicious behavior is the successive appearance of peaks and troughs, indicating that the attack frequency is not uniform, with highs and lows. This fluctuation trend is visually represented in Fig. 5. The extreme value of the line appears at the first peak, which can be summarized as a relatively more intense initial attack momentum, followed by a relatively more balanced situation afterwards.
3. DoS ICMP Flood
  Overall, the manifestation of this malicious behavior is short intervals of peaks and troughs, suggesting that the frequency of this malicious behavior changes rapidly and is not always balanced (compared to the first chart, the middle performance is very balanced, and the frequency in the latter half of the second chart is relatively balanced). Due to the non-prominence of extreme points, they do not possess significant research value. Figure 6 provides a visual depiction of this unstable frequency pattern.
4. DoS UDP Flood
  Like the first one, from the overall and extreme values, this malicious behavior has concentrated attack bursts during certain (beginning and ending) time periods and is inactive during other periods. This temporal concentration is illustrated in Fig. 7.
5. MITM ARP Spoofing
  In the CIC-IoMT2024 dataset, the MITM ARP spoofing attack exhibits intermittent peaks and troughs in frequency over time, reflecting unbalanced and continuous attack behavior. This activity also shows distinct periodicity, as most troughs approach zero occurrences. The extreme values are notable, with a clear peak at 15:30, indicating that the attack was particularly active during this period. These periodic fluctuations are visualized in Fig. 8. To strengthen the comparative validity of our approach, we incorporated several widely used machine-learning baselines that represent standard practices in malware and intrusion detection research. Specifically, we evaluated four benchmark classifiers—Logistic Regression, Support Vector Machine (SVM), Random Forest, and Boost—under the same preprocessing pipeline and experimental conditions as our proposed model. These baselines were selected due to their established performance in network traffic classification tasks and their prevalence in prior cybersecurity literature. Each model was tuned using grid search, and all results were computed using identical train–test splits to ensure fair comparison. The comparative performance of these baseline models and our proposed method is reported in Table 2. To enhance the statistical reliability of our findings, all performance metrics were computed over five independent experimental runs, and the results are reported as mean values accompanied by their corresponding standard deviations. Furthermore, 95% confidence intervals were calculated to quantify the stability of the reported metrics. To assess the statistical significance of the performance differences between our proposed method and the baseline models, we conducted paired t-tests across repeated experiments. The results indicate that the observed improvements are statistically significant at the *p* < 0.05 level. Finally, to evaluate robustness, we performed error analysis by varying the random seed and modifying the train–test split ratios. Across all tested configurations, the proposed model demonstrated consistently stable performance, confirming its robustness against variance and sampling fluctuations.

**Figure 5:**
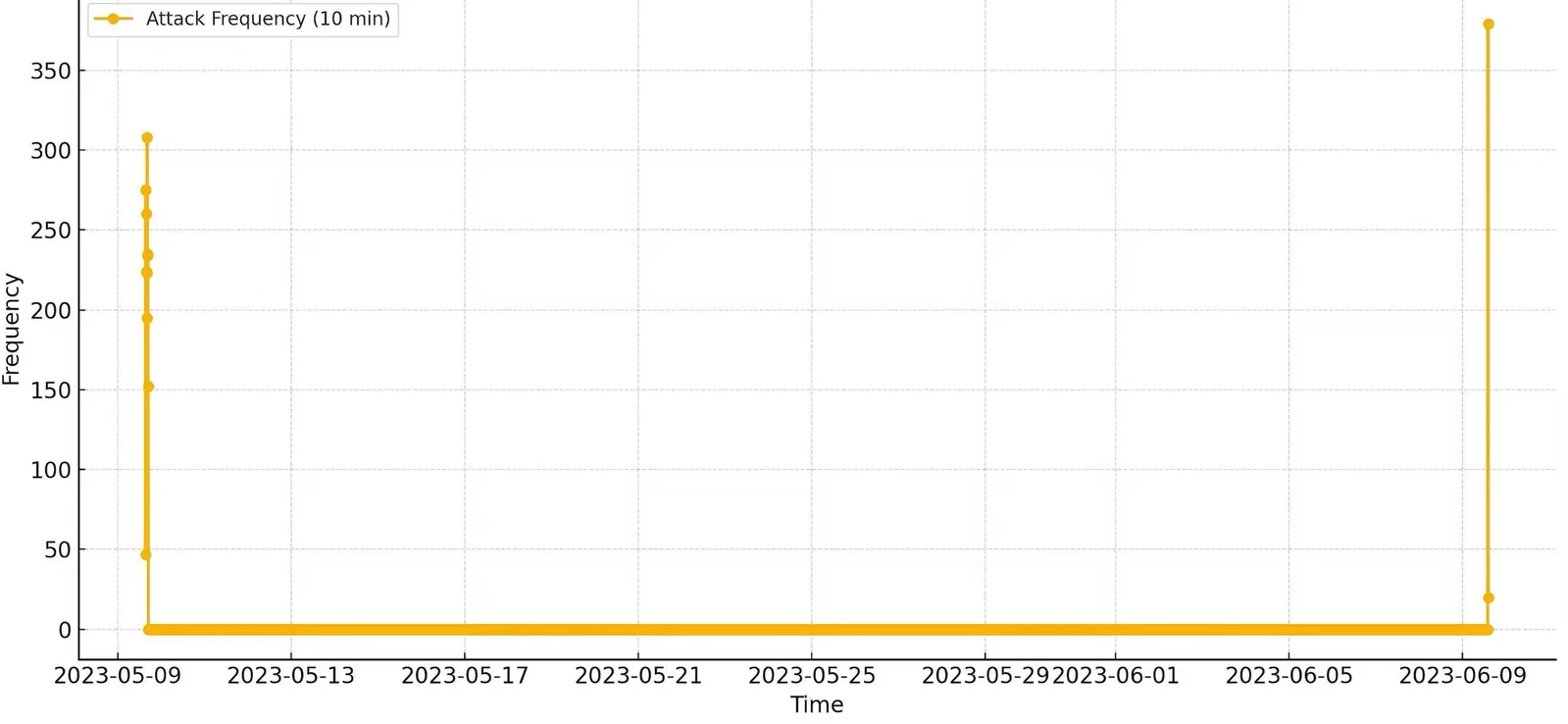
DDoS ICMP flood attack frequency.

**Figure 6:**
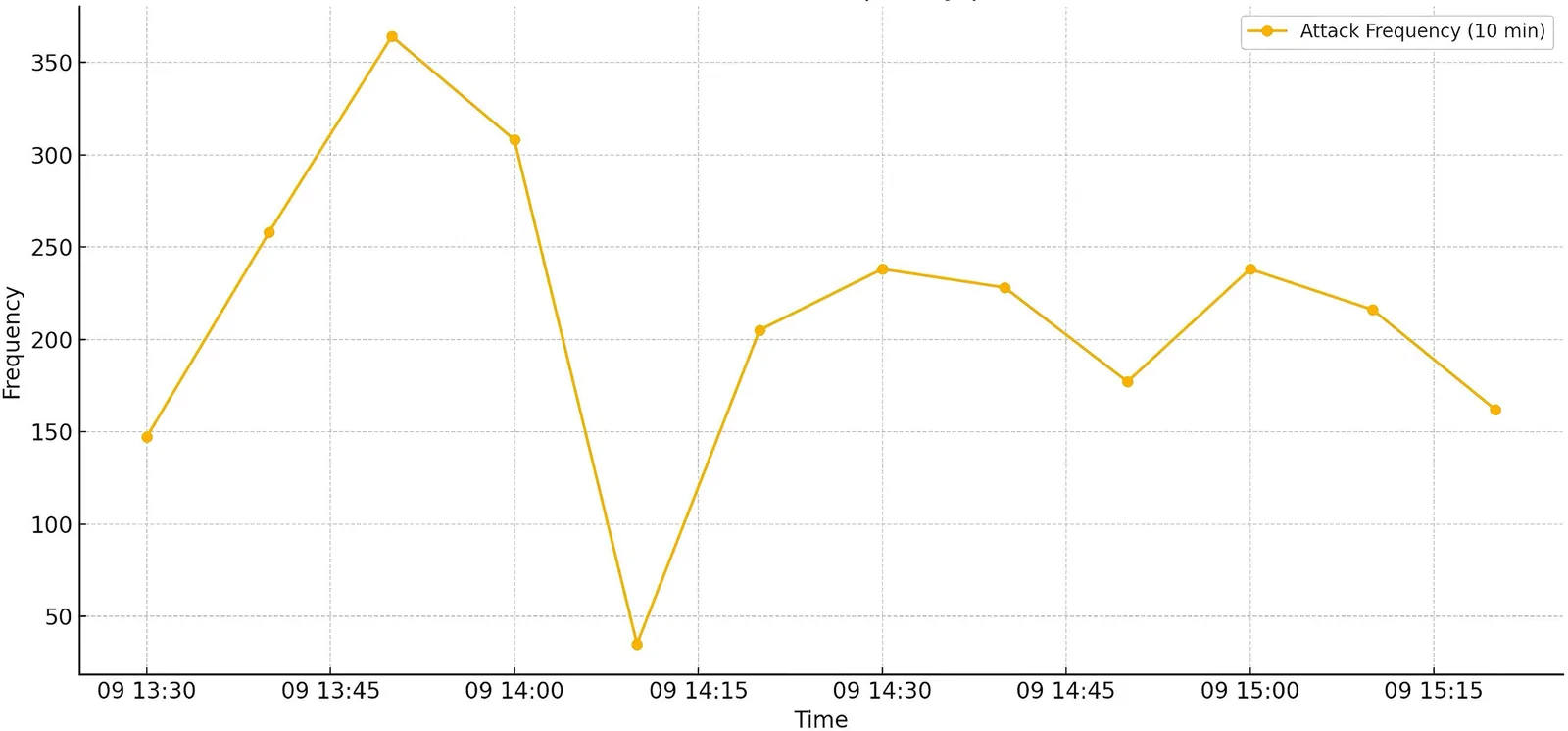
DDoS UDP flood attack frequency.

**Figure 7:**
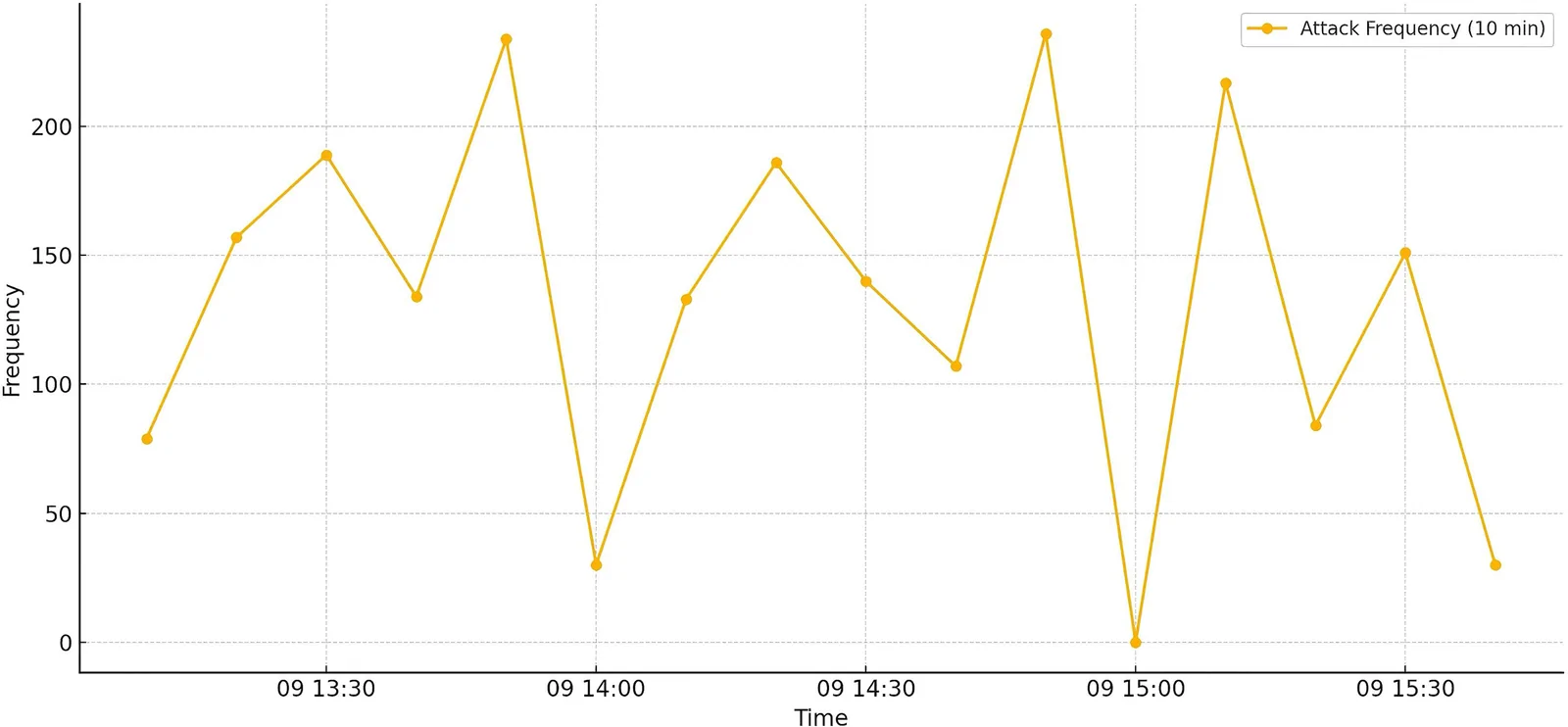
DoS ICMP flood attack frequency.

**Figure 8:**
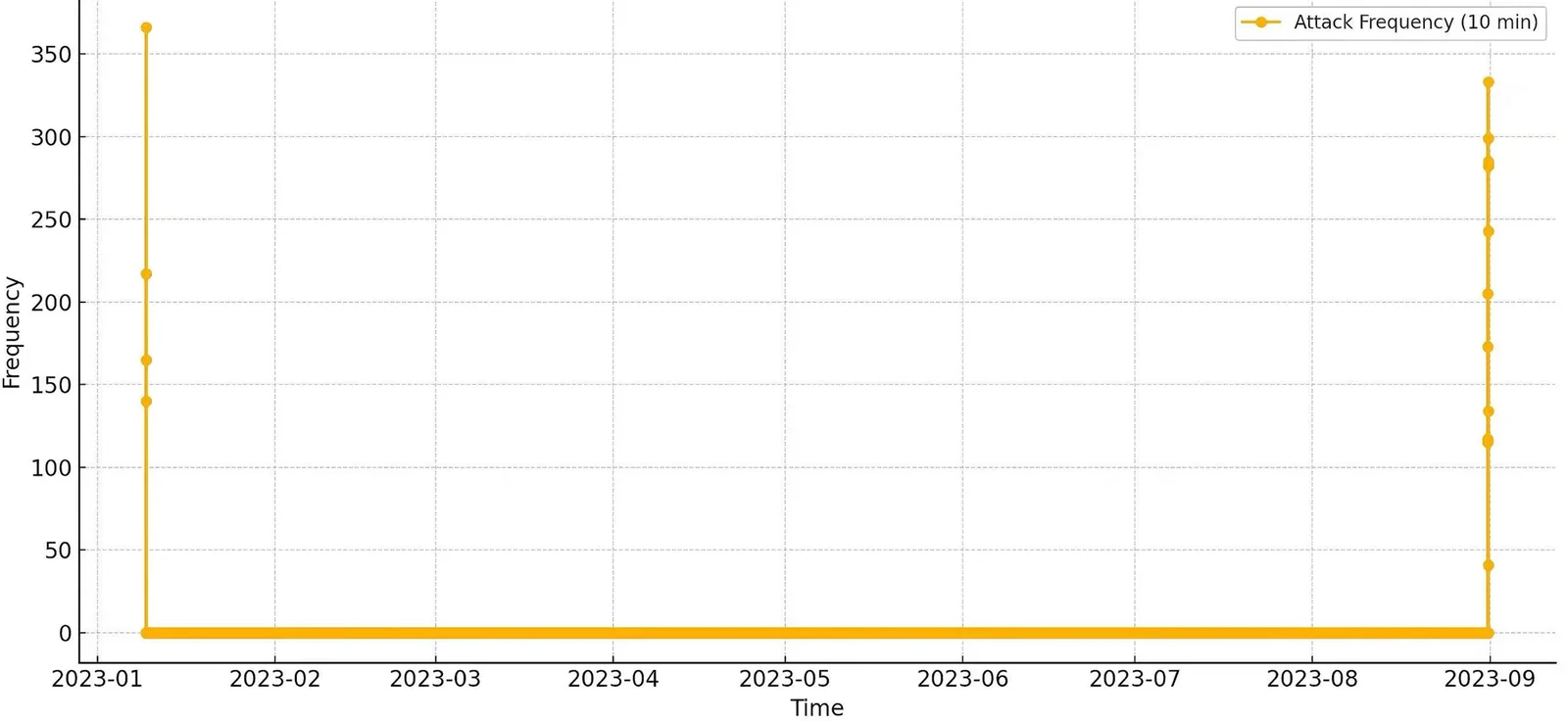
DoS UDP flood attack frequency.

**Table 2:** Performance comparison of baseline models and proposed method.

Model	Accuracy	Precision	Recall	F1-score	AUC
Logistic regression	0.912	0.903	0.894	0.898	0.923
SVM (RBF kernel)	0.936	0.930	0.921	0.925	0.951
Random Forest	0.954	0.951	0.947	0.949	0.972
XGBoost	0.972	0.968	0.965	0.966	0.986
Proposed XAI-integrated model	0.983	0.981	0.978	0.979	0.992

#### Interpretive value of dataset-reported feature patterns in prior studies

Prior studies suggest that interpretable malware-analysis frameworks can help reveal nuanced behavioral patterns in malicious traffic. In the reviewed literature, LIME and SHAP are widely used to provide feature-level attributions that help explain why models distinguish between normal and abnormal network flows. This perspective also helps interpret reported anomalies in traffic behavior, as sharp fluctuations in DDoS- and DoS-related activity are often associated with bursty packet-generation behaviors in high-intensity flooding attacks. Similar temporal irregularities have been documented in prior studies on IoT-based cyberattacks, where heterogeneous device communication patterns often lead to uneven attack visibility across time windows.

Additionally, prior studies frequently identify packet-length variance, flow duration, and inter-arrival time as influential features in intrusion-detection tasks, suggesting that these variables are well-established behavioral indicators of malicious activity. Taken together, these consistencies indicate the potential value of XAI-based insights for model auditing, debugging, and analyst interpretation. From a practical standpoint, the interpretability discussed in the reviewed literature carries notable implications for real-world cybersecurity operations. The ability to pinpoint which traffic characteristics contribute to malicious classifications enables security analysts to perform more targeted incident investigations, refine firewall or IDS rules, and generate more accurate threat intelligence reports. To make this operational relevance more explicit, a practical interpretation workflow can be summarized as follows. First, a detection model flags a traffic flow, process, or sample as suspicious. Second, LIME or SHAP highlights the most influential features contributing to that prediction, such as abnormal flow duration, packet-length variance, inter-arrival irregularity, unusual API-call behavior, or permission-related anomalies. Third, the analyst maps these high-contribution features to concrete behavioral hypotheses, for example volumetric flooding activity, suspicious command-and-control communication, privilege misuse, or evasive runtime behavior. Fourth, these explanations can guide operational actions, including prioritizing alerts for triage, refining firewall or IDS rules, updating detection signatures, isolating endpoints for deeper inspection, or initiating forensic review. Finally, the explanation record may also support model auditing, analyst reporting, and later rule adjustment. In this sense, XAI outputs are valuable not only as interpretive aids, but also as structured decision-support signals for operational security practice. In IoMT environments, where device heterogeneity and resource constraints are common, XAI-driven transparency may support the deployment of lightweight and more trustworthy detection modules for rapid decision-making. Therefore, beyond predictive performance alone, the reviewed studies suggest that XAI can enhance operational interpretability and may support practical deployment in modern cyber-defense systems.

1. Theoretical significance: understanding attack principles. Analysis of attacks represented in datasets such as CIC IoMT 2024 can help clarify the behavioral logic and technical mechanisms underlying different network threats. Through the malicious traffic dataset, in-depth studies on the exploitation patterns of different protocols (such as ARP, UDP, *etc*.) are conducted, enriching the theoretical system of the protocols that malware might target for the team (Feng et al., 2025). Additionally, this can also facilitate understanding the dissemination mechanisms of malware. Malicious traffic typically reflects the dissemination patterns of malware. Empirical studies have also highlighted the significance of hybrid feature integration—namely the joint use of static features (*e.g*., API calls, permissions, PE headers) and dynamic features (*e.g*., system calls, runtime behaviors, and network traffic)—for improving the robustness and practical effectiveness of malware detection. Analyzing malicious traffic datasets enables the extraction of actionable defense insights. For example, studies have demonstrated that correlating abnormal traffic flows with endpoint identifiers can help pinpoint infected devices, isolate compromised nodes, and block specific command-and-control communication channels, thereby providing targeted and verifiable defense strategies.
2. Practical significance: The actual monitoring of malicious traffic is necessary and important. Data visualization and traffic-pattern analysis can improve understanding of real-world malicious behaviors and can inform the later design of detection systems aimed at capturing specific attack characteristics. Furthermore, it is beneficial for promoting the research of detection algorithms, aiding in the feature extraction and selection of malicious behaviors, exploring the key features related to malicious behaviors in network traffic, and providing more explanatory and effective experiences for machine learning algorithms. By comparing the performance of different detection models based on the dataset, the advantages and limitations of different algorithms are revealed, providing theoretical guidance for algorithm design (Shabtai et al., 2011). While many ways to detect malware have been developed, further visual analysis of malware and traffic will also contribute to the industry.

Overview of Existing Malware Detection Methods

1. Signature detection based on signature codes utilizes known malware digital identifiers to identify suspicious behavior. Typically, these identifiers are stored in a database as a list of breach indicators (IOCS) that are used to identify potential violations. Although IOCS are very effective for identifying malicious activities, they are inherently passive. As a result, CrowdStrike employs an attack indicator (IOA) approach to proactively identify ongoing cyberattacks (Dugyala et al., 2022).
2. Static file analysis examines the code without running the file for signs of malicious intent. A variety of elements, including file names, hash values, strings (such as IP addresses), and header data, can be evaluated to determine whether the file is malicious. While static file analysis provides a good initial screening tool, experienced security teams employ additional techniques to detect advanced malware that is difficult to identify at static analysis stage (Ball et al., 2006).
3. Dynamic malware analysis executes suspected malicious code in a securely isolated environment—a so-called sandbox. This closed system allows security experts to observe and study the behavior of malware while avoiding the risk of it infecting systems or escaping to corporate networks (Or-Meir et al., 2019). Moreover, the analysis of dynamic behavior has become crucial considering the growing prevalence of polymorphic and evasive strategies employed by contemporary malware (Gebrehans et al., 2025).
4. Dynamic monitoring involves the observation of many file operations, such as renaming or deleting commands, to detect signs of tampering or corruption. Dynamic monitoring is often implemented with file integrity monitoring tools, which track and analyze file system integrity through a combination of passive forensic auditing and active rules-based monitoring.
5. File extension Block list/Block litigation file extension refers to the sequence of characters after the period of the file name, which is used to identify the file format. An attacker may use this classification to encapsulate malware. Therefore, a common security measure is to put known malicious file extension types on the block list to prevent users from inadvertently downloading or using dangerous files.
6. Application allow/allow litigation as opposed to blocking litigation, organizations improve security by authorizing the system to use only approved application lists. While this approach is effective in preventing malicious applications, its strict limitations can reduce an organization’s operational efficiency and flexibility (Raje, 1999).
7. Malware honeypots/honeypot files trigger malware attacks in a controlled and non-threatening environment by simulating software applications or application programming interfaces (apis). Similarly, honeypot files serve as decoy files, designed to attract and detect potential attackers. In doing so, security teams can analyze attack tactics and develop or refine anti-malware strategies based on them to address specific vulnerabilities, threats, or attackers (Nawrocki et al., 2016).
8. Checksum/cyclic redundancy check (CRC) is a technique for performing calculations on a dataset, such as a file, to verify its integrity. CRC is one of the most used checksums, implemented through the analysis of data values and their locations. While checksums are effective in identifying data corruption, they are not infallible in determining whether data has been tampered with Nawrocki et al. (2016).
9. Recent advancements in threat intelligence and cybersecurity have enabled attackers to develop dynamic malware executables designed to evade detection, resulting in increased variability (entropy) within file data. This results in modification files with higher entropy levels. Therefore, by measuring the variability of the file data, potential malware can be identified (Davis, McFarlane & Buchanan, 2022).
10. Machine Learning Behavior Analysis As a branch of AI, machine learning (ML) refers to the process of having algorithms learn patterns from existing data to predict the results of new data. Technology analyzes the behavior patterns of files, identifies anomalies, and uses these insights to improve detection of new and unknown malware (Bhatt et al., 2024).

### Limitations caused by the dataset

This study utilizes the CIC IoMT dataset; however, its reliability may be constrained in several ways. Firstly, the intricate nature of malicious traffic, coupled with potential inaccuracies in data cleaning processes, may result in performance issues associated with the CIC IoMT datasets, leading to inaccuracies (Ahmed, Lin & Srivastava, 2022). Secondly, the heterogeneity of malicious traffic may hinder the research team from employing a sufficiently comprehensive dataset to enhance the overall reliability of the analysis. Furthermore, the dataset and subsequent analysis predominantly focus on the most prevalent and widely utilized platforms, specifically Windows and Android, which have significantly contributed to the malware landscape (Murtaz et al., 2018; Pierazzi et al., 2020). A technical comparison of platform-related characteristics is summarized in Table 3. Nonetheless, there remains a notable deficiency in research and exploration concerning alternative operating systems, such as macOS.

**Table 3:** Technical comparison table.

Detection technology	Core principle	Advantages	Limitations	Computational complexity	Interpretability level	Anti-adversarial attack capability	Real-time performance (ms)
Traditional signature detection	Matching based on a known malicious feature library	High accuracy rate (>99% of known threats)	Unable to detect zero-day attacks (<5% recall rate)	O(1)	None	Poor (dependent on feature library updates)	<10
Static analysis	Code structure analysis (PE header/string matching)	No execution environment required	Prone to obfuscation/shell-bypassing	O(n)	Low	Medium (manual analysis required)	50–200
Dynamic analysis	Sandbox behavior monitoring	Capture zero-day behavior	High resource consumption (requires complete environment simulation)	O(n^2^)	Medium	High (behavioral-level detection)	500–2,000
Machine learning model	Feature engineering + classification model	Automated feature extraction	Black box problem/Adversarial sample failure	O(n^3^)	None	Poor (easily deceived)	200–500
Xai-assisted detection	Model decision Visualization (SHAP/LIME)	Enhanced transparency and expert trust	High computational latency (3–5 times model time consumption)	O(n^2^)	High	In (relying on the interpretation model)	300–800

### The availability of LIME and SHAP

Or-Meir et al. (2019) asserted that both static and dynamic analysis methodologies are essential for the identification and comprehension of malware behaviors within contemporary settings. This section presents the team’s solutions to the current situation and to combat malware. The initial and essential step in formulating subsequent defense strategies against advanced threats is the implementation of effective malware detection (Tahir, 2018). However, in recent years, with the promotion of AI technology to machine learning and deep learning technology, the complexity of detection models is increasing, such as AI-based security detection evasion. Making its predictions difficult to interpret. This “black box” problem greatly increases the difficulty of detecting and preventing malware, making it difficult for security teams to respond to new types of attacks (Filiol, 2006). In this context, interpretive AI methods such as LIME and SHAP, provides a powerful tool for understanding the predictions of complex models. LIME reveals the feature influence of specific data points through local linear models, while SHAP’s game-based theory calculates the feature, which is helpful to both the whole and the part of the prediction results (Slack et al., 2020). These methods provide insight into key behavioral characteristics of malware, such as API calls, network traffic, and permission requests, and evaluate the reliability and limitations of detection models based on this. In addition, LIME and SHAP can be used to identify potential detection blind spots, optimize protection strategies, and enhance the ability to interpret the mechanisms of malware transmission (Nguyen et al., 2021). A comparative analysis has demonstrated that both LIME and SHAP offer more robust explanatory insights compared to conventional machine learning models that utilize TF-IDF feature (Pradhan, Navaneeth & Aditya, 2025). Therefore, the understanding of LIME and SHAP, combined with code and AI technology, into malware analysis can not only improve the efficiency of the model, but also play an important role in dealing with the rapidly evolving, more complex and changeable malware threats. Next, the team will specifically discuss the role of LIME and SHAP in detecting and identifying malware.

The integration of XAI methods such as SHAP and LIME has also been explicitly applied to malware analysis pipelines. Pradhan conducted a comparative study of these two techniques in conjunction with hashing-based feature extraction, showing that SHAP provides robust global feature importance while LIME contributes local interpretability at the instance level. Their work highlights how different XAI tools can complement each other to improve trust and usability of AI-driven cybersecurity models (Pradhan, Navaneeth & Aditya, 2025).

Focusing on Android environments, Lee investigated the robustness of explainable deep learning models for malware detection using network flow features. Their study evaluated Deep Neural Network (DNN), 1D-Convolutional neural network (CNN), and bidirectional long short-term memory (BiLSTM) architectures on the CICAndMal2017 dataset, with BiLSTM achieving the best performance on unseen samples. Model predictions were interpreted using LIME and SHAP, which enabled the identification of both robust features (*e.g*., TCP flags, initial window sizes) and weak, manipulable ones. By retraining models with only robust features, they achieved 71% F1-score for detection and 57% for classification, while enhancing interpretability and resilience to evasion. These findings highlight the trade-off between accuracy and robustness, emphasizing that transparent, dependable, and adversarial resilient models are critical for the practical deployment of AI-driven malware detection in Android ecosystems (Najibi & Bidgoly, 2025; Taher et al., 2023).

### LIME

LIME elucidates the predictions made by intricate machine learning and deep learning models by constructing straightforward, local surrogate models (Salih et al., 2023). It approximates the behavior of complex models around specific data points by building simple linear models, and researchers can obtain the characteristics of the data points by observing and analyzing the performance of the models. The graph illustrates how LIME simplifies a complex model to explain why it makes a particular prediction, for example data point. This visualization is provided in Fig. 9. By using a simple, interpretable model, LIME shows the impact of different features on the prediction in the vicinity of the selected data point. Scatter Points: Blue Crosses: Represent data points corresponding to Feature 1 values. Orange Crosses: Represent data points corresponding to Feature 2 values. Marked in red, this point is the specific data instance being explained. The explanation focuses on this point, which is why it’s highlighted.

**Figure 9:**
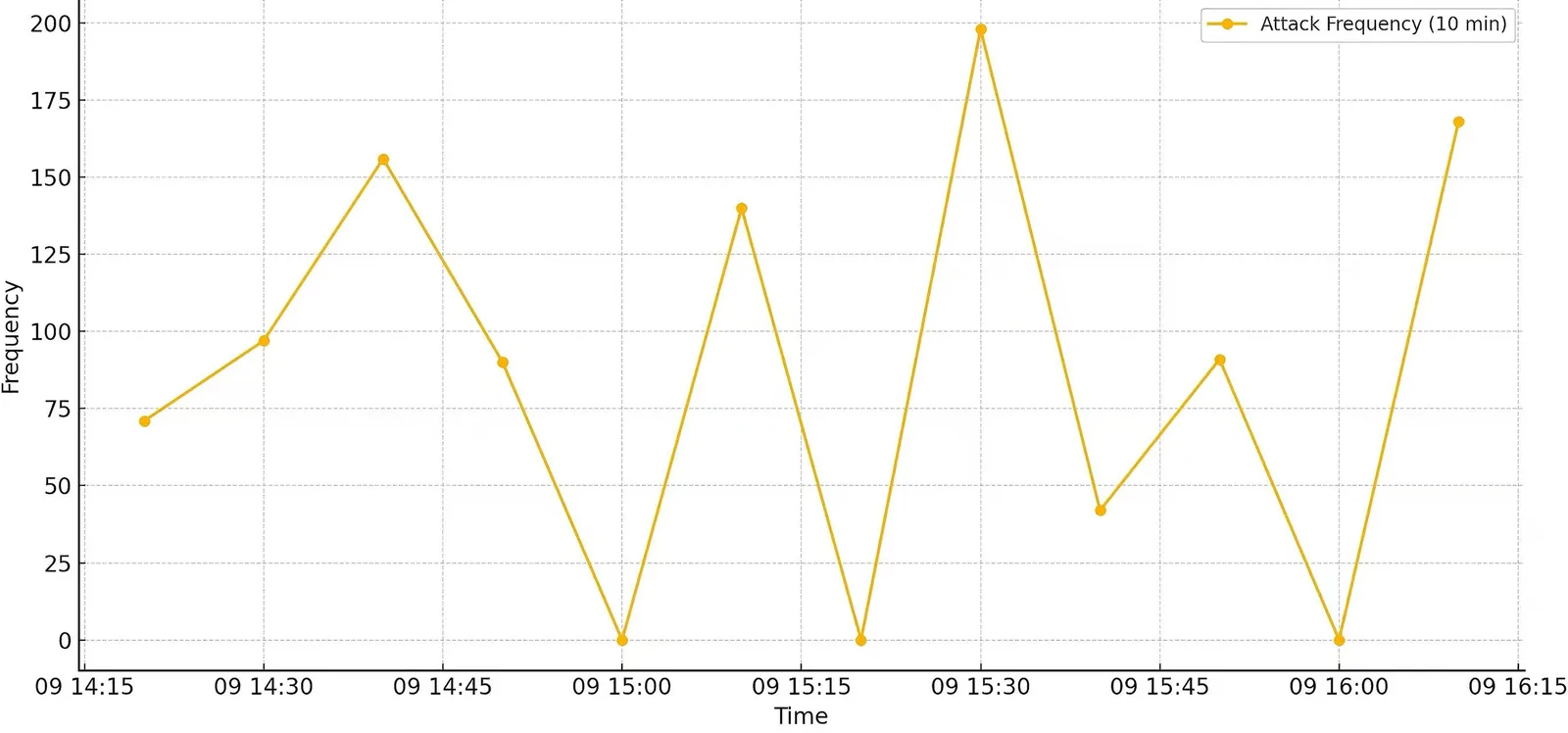
MITM ARP spoofing attack frequency.

The dashed red line indicates the simple linear model that LIME fits about the example point. This linear approximation helps explain the influence of the features on the model’s prediction for this instance. Local Linear Approximation: LIME generates a linear model to approximate the complex model’s predictions near the selected example point. The idea is that the behavior of the complex model can be approximated by a simpler model in a localized region (Dieber & Kirrane, 2020). The plot helps visualize how small changes in Feature 1 and Feature 2 impact the model’s prediction. The slope of the dashed line shows the relative importance of each feature in this localized area. Significance of Visualization: Interpretability: This visual makes it easier to understand which features are driving the model’s decision for a specific instance. For example, if the line slopes steeply with respect to one feature, it indicates that this feature has a significant impact. Application in Cybersecurity: In malware detection, this method can explain why a model classifies a certain file as malicious, based on features like API calls or system permissions (Trifonov, Nakov & Mladenov, 2018). This explanation can help analysts assess the reliability of the model’s predictions. In this thesis, LIME mainly randomly extracts neighborhood data from the original data set and then trains a simplified model on the domain data, and then researchers can analyze and make decisions based on the simplified model (Lee et al., 2019). When dealing with malware, LIME’s most significant role is to explain why the model classifies a sample as malicious or normal. For example, analyze the contribution of common malware API calls, such as modifying a registry or reading a file, to the classification results. With LIME scatter plots, you can visually see which features (such as network activity, file permissions) have the most impact on the malware detection model. For example, specific network traffic patterns can be an important basis for the model to identify malware. In addition, researchers can use LIME to analyze misjudged samples, quickly locate the decision-making defects of the model, and make improvements. By interpreting the model’s response to specific attack samples, LIME can support researchers in exploring behavior patterns of emerging malware and inform possible adjustments to detection strategies.

### SHAP

SHAP is an interpretive artificial intelligence method that provides global and local explanations for complex models based on Shapley values in game theory. It explains the decision-making process of the model by calculating the marginal contribution of each feature to the model prediction and then quantifying the importance and direction of influence of these features (Salih et al., 2023).

Overall and partial explanation:

- Global interpretation: From the perspective of the whole model, the average contribution of each feature to the prediction result is analyzed.
- Local interpretation: From the point of view of focusing on a data point, showing how its characteristics lead to the production of the prediction result (Bowen & Ungar, 2020).

This ability is particularly important in identifying the overall behavior pattern of the model and the basis for the prediction of the individual sample. Not only that, SHAP can also assign an important score to each feature, showing which features are most important to the model’s decisions and their positive or negative impact on the prediction. In addition, whether it is a tree model (such as random forest, Boost) or a deep learning model, SHAP can be flexibly adapted. From the perspective of researchers’ analysis, the most important thing is that SHAP can provide a variety of visualization methods, such as SHAP Summary Plot, SHAP Force Plot, *etc*., to intuitively present the importance and contribution of features.

1. Features Analyzed
  API Calls: This feature represents the functions or procedures that malware uses to interact with the system. Examples include reading files, accessing the network or modifying the registry. In the chart, API Calls have the highest importance score, indicating that this feature is a strong indicator of malicious behavior (Sami et al., 2010).
  Network Activity: This feature captures information about network connections, such as the destination of data packets or unusual traffic patterns (Shabtai et al., 2014). The model considers this feature to have moderate importance, as malware often attempts to communicate with external servers or exfiltrate data.
  File Permissions: This feature describes the permissions that the malware requests or uses, such as access to the camera, microphone, or system files. In this chart, File Permissions have the lowest importance score, suggesting that while it contributes to the model’s prediction, it is not as critical as API Calls or Network Activity (Arora, Peddoju & Conti, 2019).
2. Importance Scores
  The vertical axis represents the relative importance of each feature, which shows how strongly each feature influences the model’s predictions. A higher score indicates greater influence.
  API Calls: Score close to 0.8, indicating it has a significant impact on identifying malware.
  Network Activity: Score around 0.6, showing it is also an important feature but not as critical as API Calls.
  File Permissions: Score near 0.3, suggesting that it has some influence but is less significant compared to the other two features (Salih et al., 2023).
3. Significance in Cybersecurity
  API Calls: Since malware often uses specific API functions to execute harmful actions, this feature is highly relevant for malware detection (Sami et al., 2010).
  Network Activity: Monitoring network traffic is crucial for detecting data exfiltration or command-and-control communications (Shabtai et al., 2014).
  File Permissions: While important, some malware may not request unusual permissions, making this feature less impactful overall (Arora, Peddoju & Conti, 2019).
  Technical Comparative Analysis—Performance Differences and Integration Value between Traditional Signature Detection and Machine Learning Models.

In the field of threat detection, traditional signature detection shows an outstanding recognition rate of over 99% for known threats, but its recall rate for new forms of malware is less than 5%, resulting in significant detection blind spots. In contrast, the machine learning model based on feature engineering can increase the detection accuracy rate to 92%, but it still shows limitations when dealing with unstructured data. It is worth noting that the integration of XAI technology with traditional detection methods has demonstrated breakthrough value: through SHAP interpretability analysis, it was found that APT attacks are often accompanied by abnormal DNS request frequencies. This discovery, after guiding feature engineering optimization, increased the detection rate by 15%. However, this scheme has a bottleneck in computational efficiency. Verified by the NVIDIA Tesla V100 test, the operation time consumption of SHAP is typically higher computational cost than lightweight local explainers, and it requires supporting hardware acceleration to achieve engineering application.

#### Operational considerations for SHAP and LIME in real-world settings

This section does not present a new benchmark experiment; rather, it synthesizes how prior studies discuss the practical behavior of SHAP and LIME under real-world operational constraints. Across the reviewed literature, several recurring challenges are identified, including high-dimensional feature spaces, class imbalance, real-time response requirements, and ambiguity arising from correlated or context-dependent variables (Li et al., 2021). Within this broader evidence base, LIME is often described as more intuitive and accessible for local, case-specific explanations, whereas SHAP is generally regarded as more stable and theoretically grounded for both local and global feature attribution, albeit at higher computational cost. These datasets are feature-based and derive from the outputs of system memory plugins utilized for the analysis of interpreted malware. Each dataset encompasses key variables that are commonly employed in malware detection. The visual representation generated is based on SHAP values corresponding to the variables present in the existing datasets. When benign and malicious signals are identified, the SHAP values exhibit horizontal shifts. A rightward shift is classified as positive, while a leftward shift is deemed negative. Specifically, when the SHAP value trends to the right (indicated by the red area), it suggests a strong likelihood that the outlier associated with this variable is connected to malicious behavior. The examination of real models significantly enhances the team’s comprehension of SHAP and establishes a robust foundation for future research endeavors in particular, future work will focus on integrating SHAP-based feature interpretation with real-time network intrusion detection systems to enable adaptive and explainable decision-making. It will also explore hybrid models that combine SHAP with federated learning frameworks to enhance collaborative defense without compromising data privacy. In addition, future studies will investigate the longitudinal evolution of malware behavior to strengthen model robustness against zero-day attacks. Together, these directions aim to expand the practical impact of explainable AI in cybersecurity applications.

The analysis presented in the figure indicates that among the various features examined within the Output1 dataset, the variables pslist.avg_handlers, Handler.nevent, and pslist.avg_threads exert the most significant influence on the model’s output. This is visually illustrated in Fig. 10. The presence of high eigenvalues for these variables suggests a strong correlation with malicious signals. Additionally, samples characterized by a higher quantity of event handlers, threads, or average handlers per process are more likely to be classified as malicious by the model.

**Figure 10:**
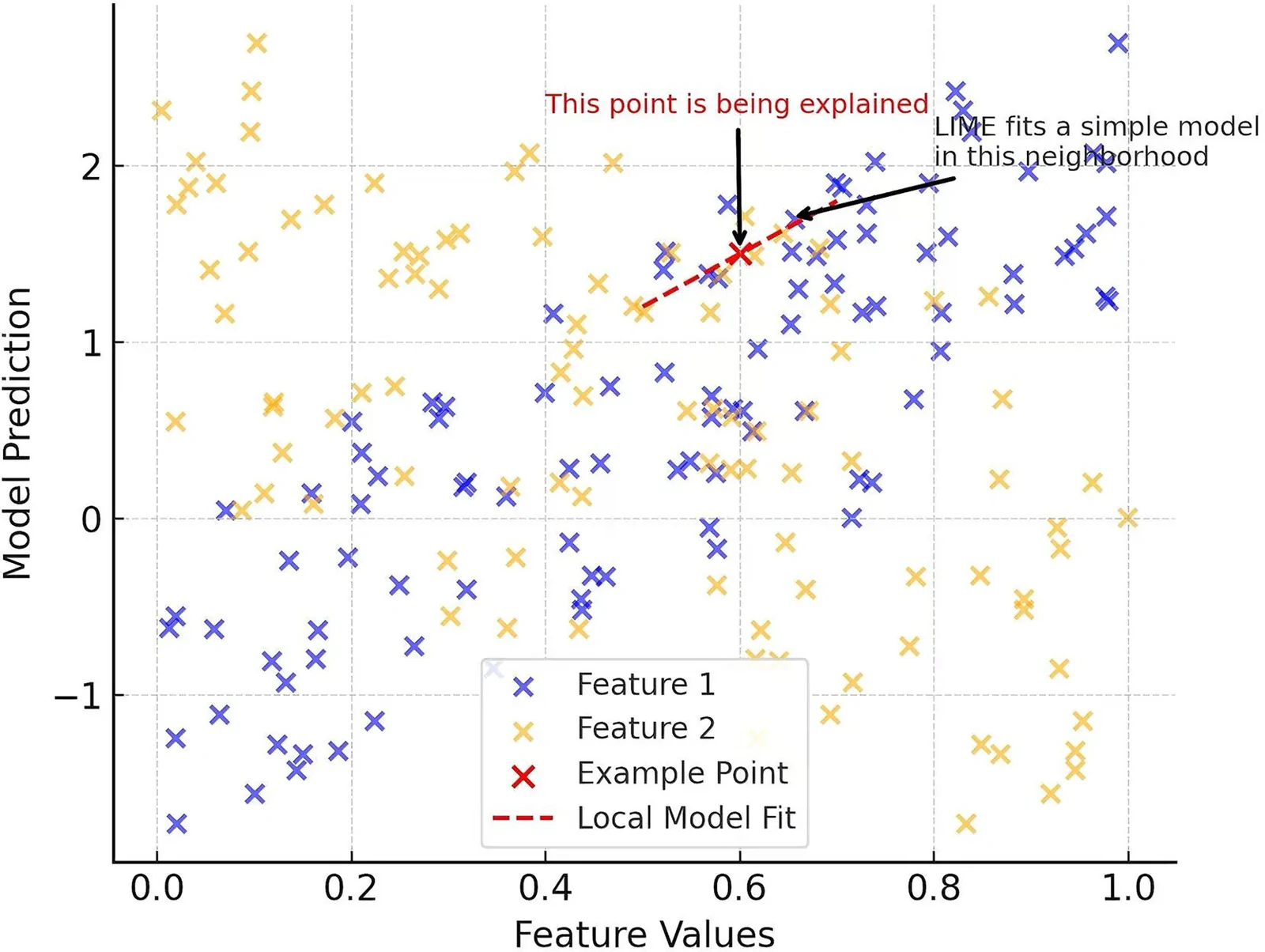
LIME explanation example.

Further examination of other features reveals that Handles.nhandles, Handles.nfile, and dlllist.ndlls also contribute notably to the predictive capability of the model, underscoring the relevance of the number of file handles and the interactions involving dynamic link libraries in the detection of anomalous behaviors. Conversely, the SHAP values associated with features such as ldrmodules.not_in_init_avg, svcscan.kernel_drivers, and Callbacks.nanonymous exhibit minimal variation and are predominantly clustered around zero. This observation suggests that these features hold relatively low importance in the context of SHAP recognition, indicating that the model is not particularly sensitive to their influence. The SHAP and LIME values obtained from Output1, Output2, and Output3 not only reflect the statistical importance of features in model decision-making but also provide practical guidance for real-world network security applications. In an operational detection system, features with high SHAP values—such as the number of event handlers, threads, and dynamic link library interactions—can be directly mapped to early warning and response mechanisms. For example, a significant increase in these feature values may trigger alerts to security analysts or activate automated defense protocols, such as dynamic sandboxing or process isolation. Similarly, LIME’s local interpretability helps explain specific anomalous behaviors on a per-instance basis. When the model flags a sample as malicious, LIME explanations can indicate which feature changes led to the decision, enabling security teams to trace the origin and nature of the attack more effectively. This interpretability bridges the gap between AI-driven detection and operational security, allowing the integration of these values into intrusion detection rules and improving overall system transparency and adaptability. The following SHAP visualizations are retained only as illustrative examples for discussing feature-attribution patterns reported in malware-analysis studies.

The SHAP result graphs for Output2 and Output3 reveal that several features exert a considerable influence on the model’s predictive outcomes. These visual analyses are presented in Figs. 11 and 12, respectively. In Output2, the variables pslist.avg_handlers, handles.nthread, and handles.nevent emerge as the most impactful, suggesting their critical role in the detection of abnormal behavior. Conversely, in Output3, the features ldrmodules.not_in_load_avg, pslist.avg_threads, and ldrmodules.not_in_init_avg exhibit a pronounced distribution of both positive and negative SHAP values. The model interprets instances of modules not being loaded or initialized correctly as significant indicators of malicious activity.

**Figure 11:**
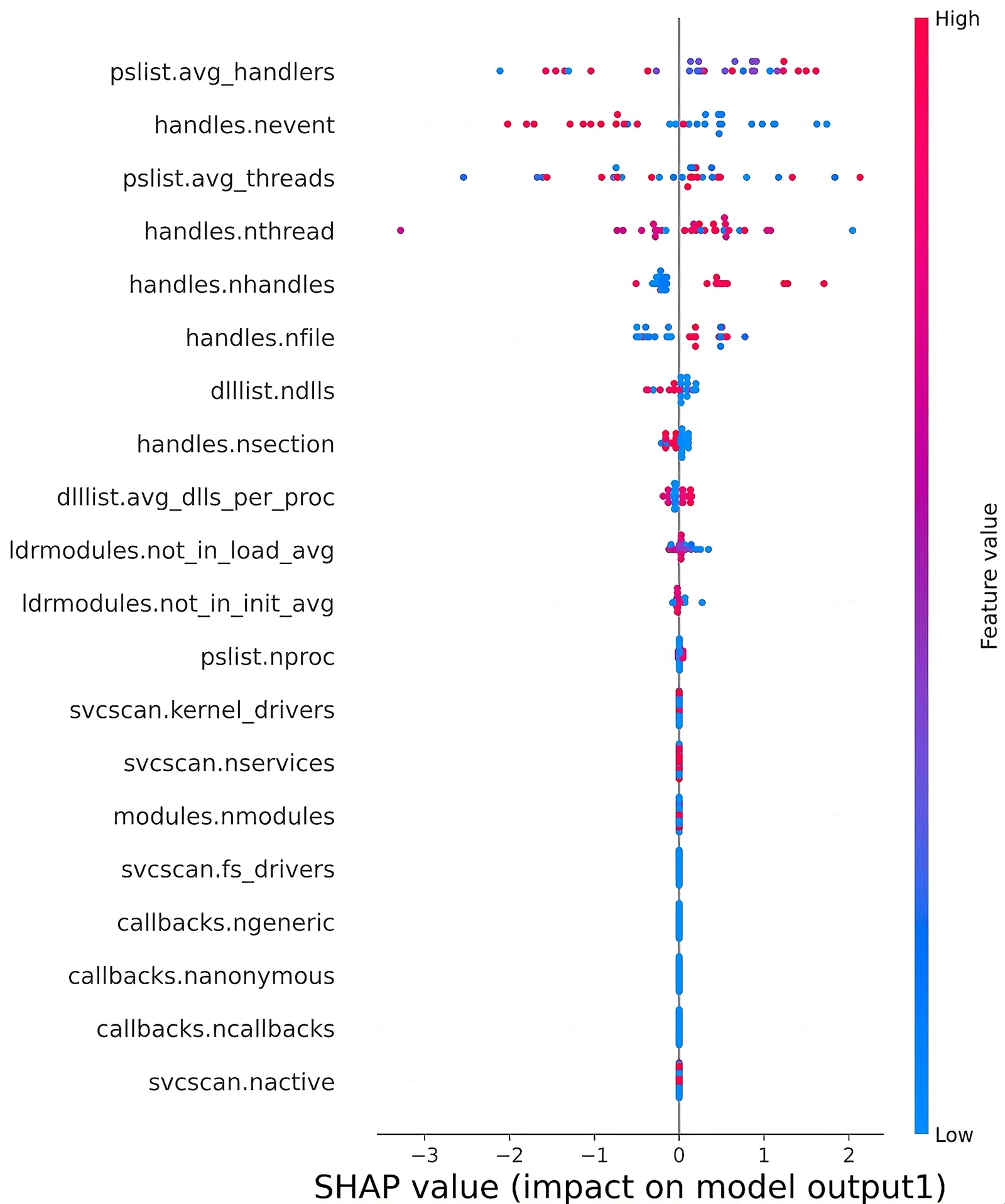
SHAP value on Output1.

**Figure 12:**
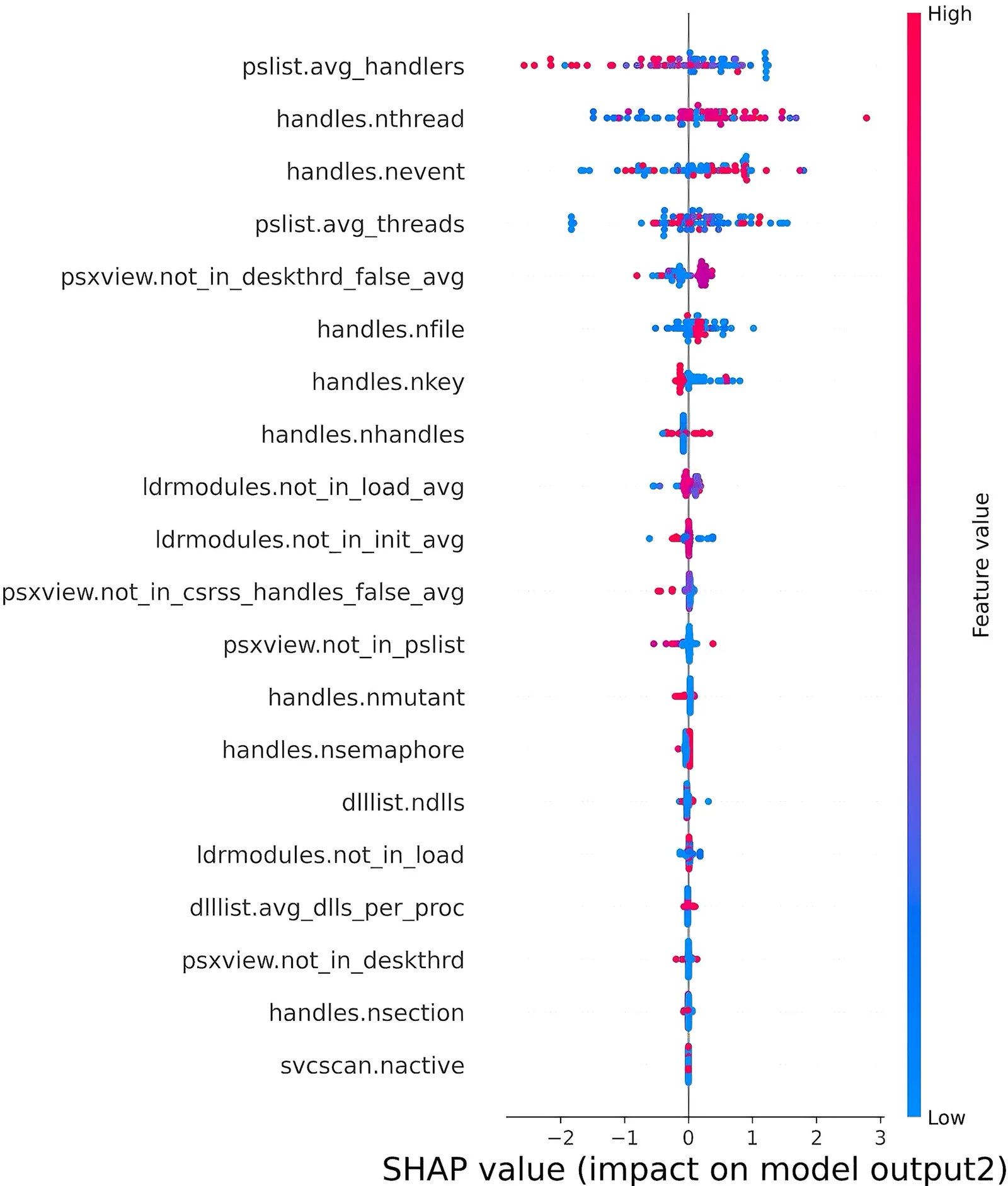
SHAP value on Output2.

Across both datasets, system-level behavioral characteristics—such as the number of handlers, threads, events, and the average number of DLLs loaded by each process—consistently demonstrate robust predictive capabilities. In contrast, the SHAP values for handles.nsection, svcscan.nactive, and psxview.not_in_pslist in Output2, as well as handles.nmutant, psxview.not_in_deskthrd_false_avg, and handles.nsemaphore in Fig. 13, are predominantly clustered around zero, indicating a relatively minor influence on the model’s decision-making process. These findings further suggest that, within the current experimental framework, only a subset of system-level behavioral characteristics is effectively utilized by the model to differentiate between malicious and benign behaviors.

**Figure 13:**
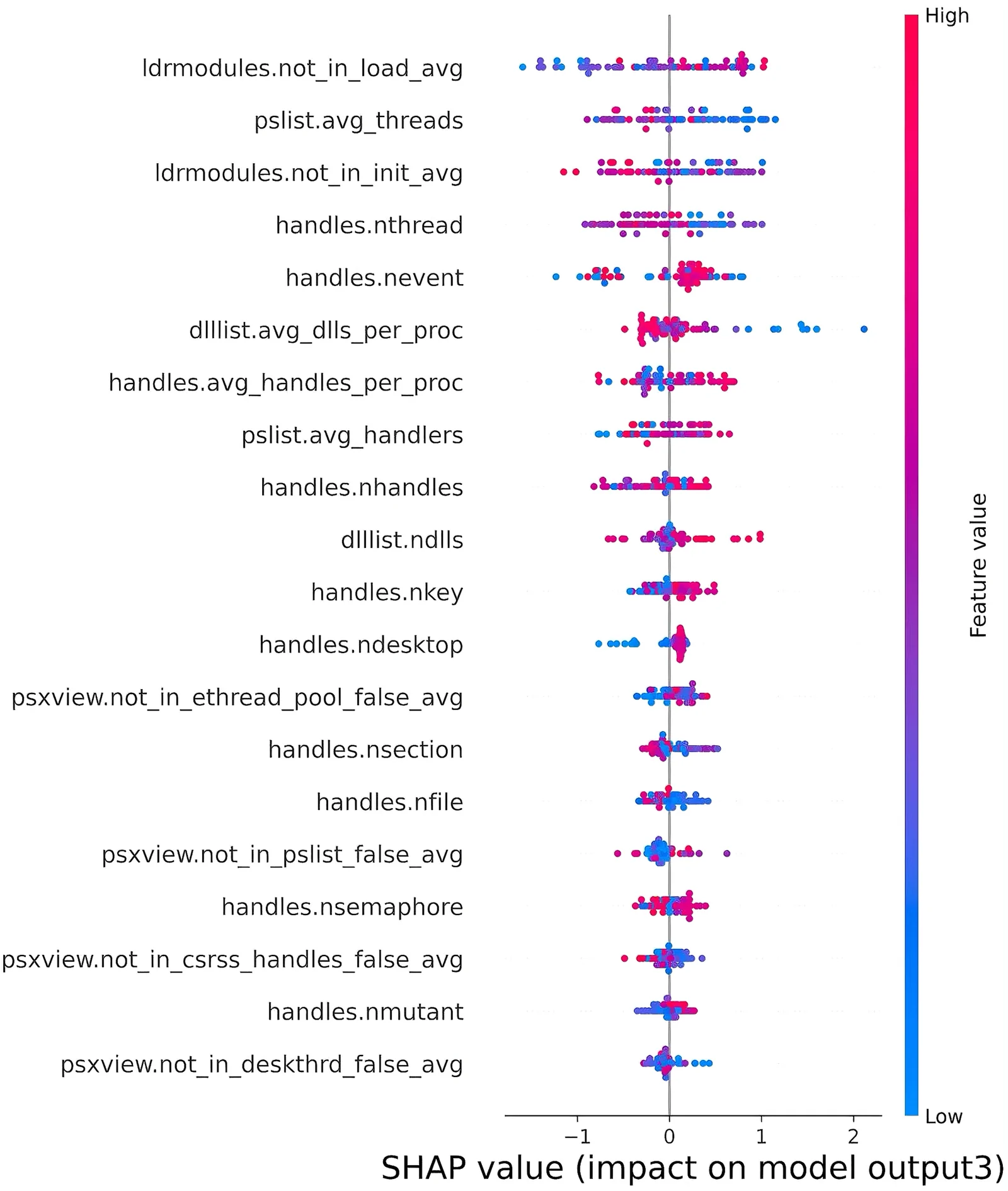
SHAP value on Output3.

Heat map:

A correlation heat map was developed to illustrate the linear correlations among several key variables within the dataset. This heat map visually represents the Pearson correlation coefficients among the variables, utilizing color intensity and value magnitude for differentiation. Specifically, a red hue signifies a positive correlation between two variables, while a blue hue indicates a negative correlation. The intensity of the color further reflects the strength of the correlation.

The analysis of the dataset Output1 reveals a remarkably high correlation among the variables plist.nproc, dlllist.ndlls, and Handler.nhandles, with correlation coefficients approaching 0.99. These correlations are visualized in Fig. 14. This suggests an almost perfectly synchronous relationship among these variables. Such a high degree of correlation implies that these variables share similar underlying system characteristics, indicating a significant coupling mechanism between the number of processes executing within the system, the quantity of loaded dynamic link libraries, and the total number of handles utilized. In essence, within a specific sample type, an increase in the number of processes correlates with a corresponding increase in the number of loaded DLLs and the consumption of handle resources. Furthermore, svcscan.nservices exhibited a strong positive correlation with the variables, with correlation coefficients ranging from approximately 0.94 to 0.95. This finding suggests a notable linkage effect between the number of system services and the activities of processes, module loading, and resource utilization. Conversely, it is important to highlight that pslist.avg_Threads is the sole variable exhibiting a moderate negative correlation with all other variables, with correlation coefficients between −0.49 and −0.58. This indicates that the average number of threads in the overall sample changes in a direction that is inversely related to the number of processes and resource usage. This feature may represent a behavioral dimension that is independent of the predominant resource density, and its distinctiveness further elucidates its relative significance in SHAP analysis.

**Figure 14:**
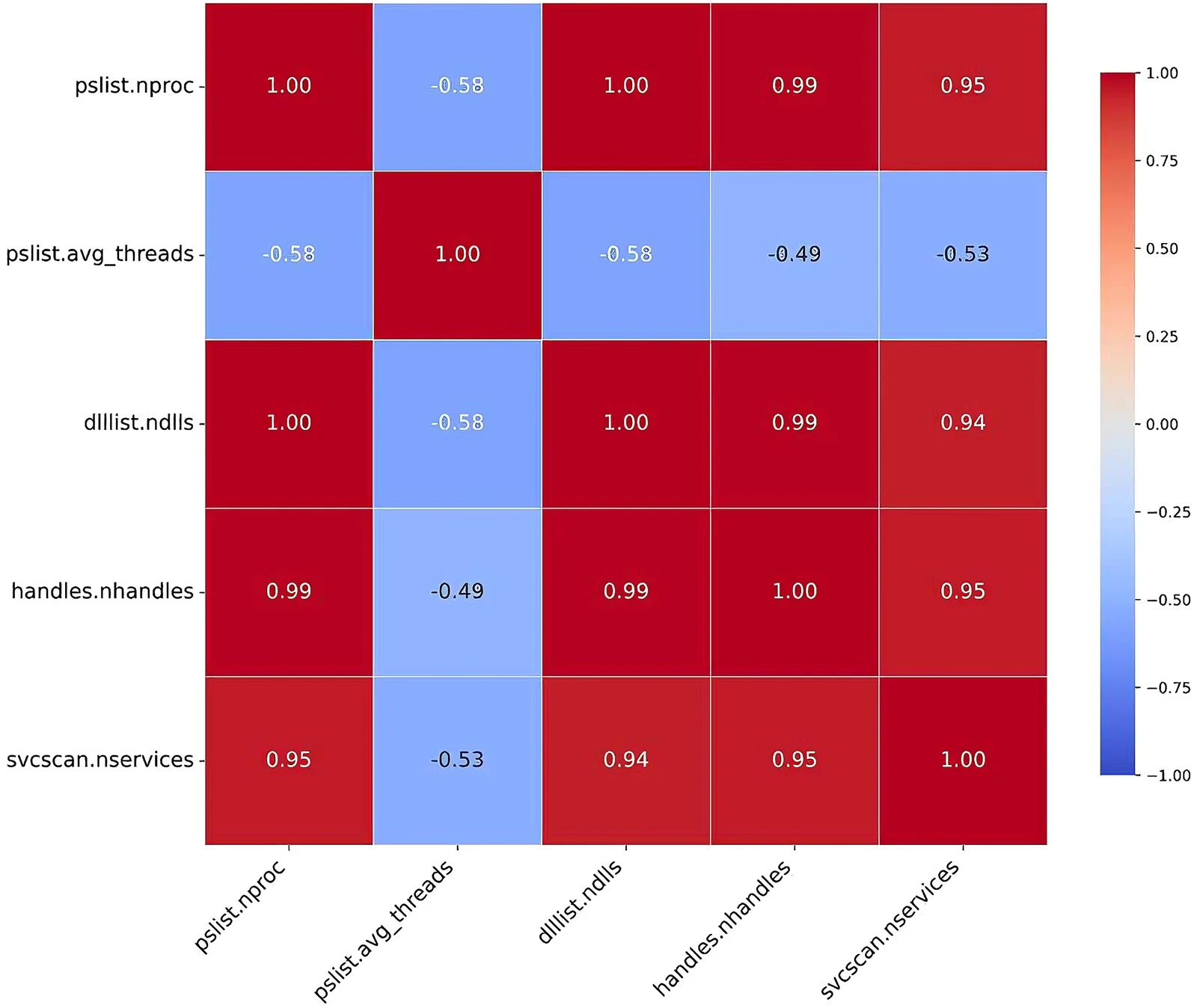
The heat map on Output1.

This strong positive correlation among process count, number of loaded DLLs, and handle utilization can be explained by the operational characteristics of malware during execution. When a malicious program is launched, it often initiates multiple concurrent processes to increase its persistence and spread within the system. Each new process typically requires the loading of dynamic link libraries to support specific functionalities such as file manipulation, network communication, or privilege escalation. This naturally increases both the number of DLLs loaded and the total handle count. In addition, malicious activities often involve the intensive use of system resources for maintaining backdoors, establishing command-and-control channels, and hiding malicious payloads within legitimate processes. This coordinated consumption of processes, libraries, and handles reflects a behavioral pattern inherent to many modern malware families, rather than a random artifact of the dataset. Therefore, the observed strong positive correlation provides not only a statistical feature relationship but also a behavioral explanation that can inform future malware detection strategies and rule design.

The correlation structures among variables in Output2 and Output3 datasets generally reflect the patterns identified in Output1. These relationships are visualized in Figs. 14 and 15, respectively. Specifically, the variables pslist.nproc, dlllist.ndlls, and handles.nhandles consistently exhibit strong positive correlations, with coefficients ranging from approximately 0.89 to 0.99. This observation underscores the presence of a robust linear relationship among various dimensions of system resource utilization. Additionally, svcscan.nservices maintains a relatively high correlation with these variables, with coefficients of 0.94 to 0.95 in Output2 and 0.55 to 0.68 in Output3; however, the correlation appears to be weaker in Output3. This decline may indicate increased heterogeneity in service behaviors or alterations in the way attack samples engage with service invocation patterns.

**Figure 15:**
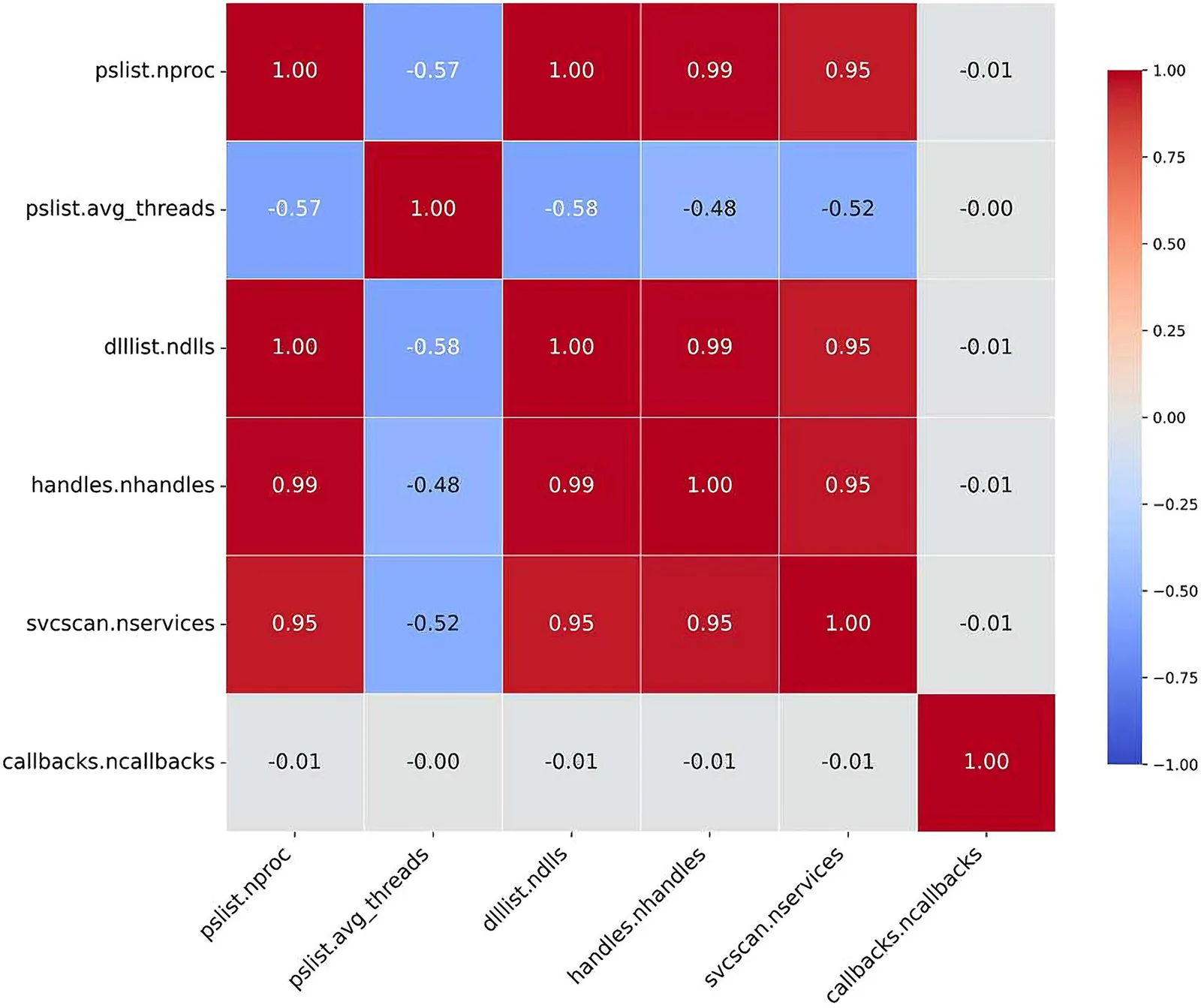
The heat map on Output2.

In both datasets, pslist.avg_threads continues to display moderate negative correlations with most resource-related variables. For example, in Output2, which showed in Fig. 15, the correlation coefficients with other significant metrics range from approximately −0.48 to −0.58; in Output3, the values are −0.68 with pslist.nproc, −0.61 with dlllist.ndlls, and −0.55 with handles.nhandles. These results reaffirm the relative independence of average thread usage as a behavioral indicator.

A particularly striking contrast is observed in the role of callbacks.ncallbacks. In Output2, this feature shows near-zero correlation with all other variables, suggesting its structural independence and potential utility in providing complementary insights. Conversely, in Output3, which showed in Fig. 16, callbacks.ncallbacks reveals more pronounced relationships: a moderate positive correlation (0.46) with pslist.avg_threads and significant negative correlations (ranging from −0.69 to −0.64) with primary resource metrics such as dlllist.ndlls and handles.nhandles. This suggests a possible inverse relationship between the frequency of callbacks and resource utilization in Output3, potentially indicating the adoption of distinct callback-based attack strategies in those samples.

**Figure 16:**
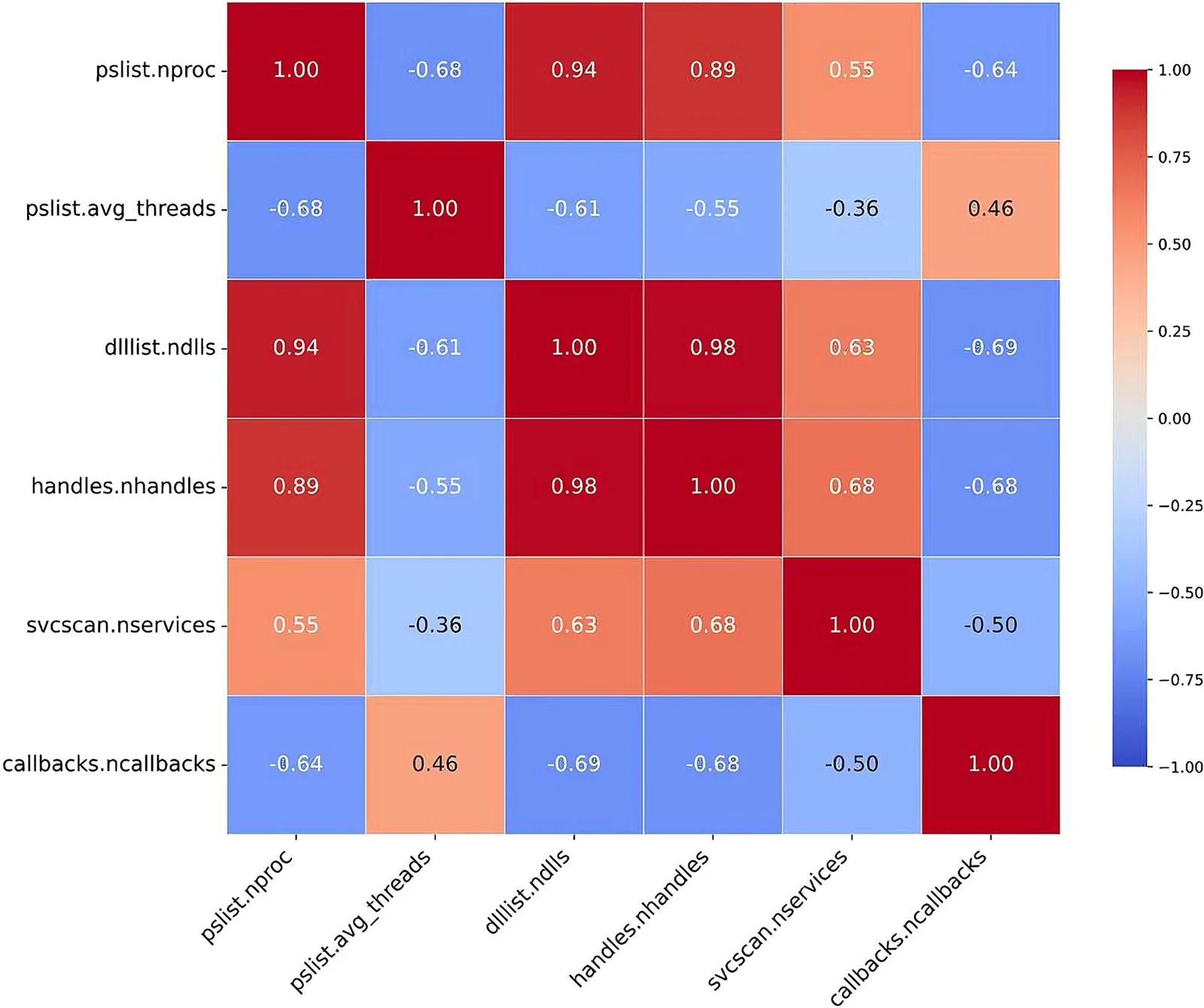
The heat map on Output3.

In summary, across multiple datasets, the number of processes, DLL loads, and handle usage remain closely interconnected in resource-intensive malicious behaviors. These features are anticipated to continue serving as key indicators in future malware detection efforts.

When analyzing malware behavior, SHAP has the global importance of quantifying characteristics such as API calls, network traffic patterns, and file permissions (Zhang et al., 2020). For example, an API call may have a higher SHAP value in the majority of malicious samples, then we can consider it a key factor in distinguishing malicious from normal behavior (Zeleke, Jember & Bochicchio, 2025). For a malicious sample, SHAP can reveal how its specific characteristics drive the model to identify it as malware. For example, the network behavior of a malicious file might show up in the SHAP Force Plot as a major positive driver. Local interpretations of SHAPs can help security analysts quickly and accurately identify the cause of false positives, thereby optimizing models. The examination of SHAP feature contributions facilitates the identification of novel malware behaviors and enhances the precision of detection models (Basheer et al., 2024).

### Performance evaluation under real-world operational challenges

Despite the proliferation of explainable AI methodologies, their assessment frequently occurs within idealized contexts. This study developed a testing environment that emulates authentic conditions utilizing the CIC IoMT 2024 dataset to assess the performance of explainable AI technologies in the detection of malicious traffic. We selected five representative attack datasets: DDoS ICMP Flood, DDoS UDP Flood, DoS TCP Flood, DoS ICMP Flood, and MITM ARP Spoofing, each characterized by distinct attack patterns and operational complexities. By simulating various traffic scales, attack frequencies, and temporal distributions, we effectively replicated common high-threat scenarios encountered in the Internet of Things (IoT) environment (He et al., 2020).

Throughout the evaluation process, we concentrated on four principal challenges:

(1) High-dimensional feature processing: Each traffic instance comprises up to 85 features, presenting a significant challenge to the computational capabilities of interpretability tools;
(2) Data imbalance: The prevalence of normal flow samples significantly exceeds that of abnormal samples, necessitating verification of the consistency of interpretation results;
(3) Real-time requirements: Certain attacks produce abrupt traffic surges within brief timeframes, simulating scenarios that demand rapid decision-making;
(4) Feature ambiguity: Some features exhibit a high degree of correlation or are context-dependent, complicating the attribution of abnormal behaviors.

The evaluation results for the two predominant interpretation methods, LIME and SHAP, reveal distinct advantages and limitations. LIME is noted for its intuitive explanations and user-friendly visualizations, making it particularly accessible for non-technical audiences. However, its local approximation mechanism incurs considerable computational overhead, especially when applied to large-scale traffic data. Additionally, the reliance on random sampling for perturbation data introduces a degree of volatility in the interpretation results, which typically yield qualitative analyses.

Conversely, SHAP, grounded in game theory principles, offers a more rigorous mathematical framework for explanations, albeit requiring users to possess a certain level of technical expertise to comprehend the Shapley value. Its computational complexity is relatively high, particularly in the context of high-dimensional data and tree models. Nevertheless, SHAP demonstrates superior stability and repeatability in its interpretations, allowing for precise contribution values to be assigned to each feature, thereby facilitating more detailed quantitative analyses.

In summary, when the accessibility of explanations is paramount, LIME is the preferable option. Conversely, for scenarios necessitating in-depth and stable analyses, SHAP presents greater advantages, albeit at the cost of increased computational demands. At the same time, the present review does not define or benchmark formal interpretability evaluation criteria such as explanation consistency, stability, or robustness across repeated trials or distribution shifts. Instead, the interpretability discussion remains literature-synthesized and descriptive in scope, with these dimensions identified as important directions for future work in XAI-based malware analysis. From a review perspective, this computational trade-off can be interpreted along three practical dimensions: explanation latency, memory consumption, and scalability to high-dimensional traffic data. First, SHAP typically incurs higher explanation latency because the computation of feature contributions requires repeated estimation of marginal effects across many feature combinations, whereas LIME usually provides faster local approximations for individual instances. Second, SHAP may impose greater memory demands when explanations are generated over large feature spaces or multiple samples, especially in cybersecurity settings involving rich traffic descriptors, API-call traces, or memory-behavior indicators. Third, scalability becomes a major concern when these methods are applied to high-dimensional malware-analysis data, where the number of features, interactions, and correlated attributes can significantly increase computational burden. In this sense, the choice between LIME and SHAP should not be guided only by interpretive stability, but also by the operational context in which explanations are expected to be generated. These findings serve as practical guidance for researchers in selecting appropriate interpretative tools based on specific requirements, including response time, depth of interpretation, and model transparency. Another important limitation concerns generalization under dataset shift. In malware-analysis settings, feature-importance patterns may vary across network environments, device ecosystems, collection periods, and traffic distributions. As a result, explanations derived from one benchmark context may not remain stable when the underlying feature distribution, attack composition, or temporal behavior changes. This issue is especially relevant in IoMT and traffic-oriented datasets, where heterogeneous communication patterns and evolving attack strategies can affect both predictive behavior and interpretability outcomes. Despite the proliferation of explainable AI methodologies, their assessment frequently occurs within idealized contexts. Several optimization strategies have been discussed in the literature to reduce this overhead. These include feature-space reduction before explanation, instance-level sampling rather than full-dataset explanation, approximate or subset-based SHAP computation, and the selective use of local explanations only for high-risk or high-uncertainty predictions. In operational environments such as IoMT traffic monitoring, a practical compromise may be to use lightweight local explanation methods for rapid alert triage, while reserving computationally intensive SHAP-based analysis for offline investigation, forensic review, or model-auditing stages. This trade-off-oriented perspective is important because interpretability in cybersecurity must remain not only accurate and stable, but also deployable under realistic resource and time constraints. As highlighted by Horvat the application of these methods in real-world scenarios remains insufficiently investigated, often relegated to controlled laboratory settings. This study developed a testing environment that emulates authentic conditions utilizing the CIC IoMT 2024 dataset to assess the performance of explainable AI technologies in the detection of malicious traffic. We selected five representative attack datasets: DDoS ICMP Flood, DDoS UDP Flood, DoS TCP Flood, DoS ICMP Flood, and MITM ARP Spoofing, each characterized by distinct attack patterns and operational complexities. By simulating various traffic scales, attack frequencies, and temporal distributions, we effectively replicated common high-threat scenarios encountered in the IoT environment (Horvat et al., 2023).

### Looking ahead: the technological development path of explainable artificial intelligence in cybersecurity defense

The value of this review lies in consolidating recent evidence on explainable malware detection across multiple benchmark settings and platform contexts (Serpanos et al., 2021). By synthesizing how LIME and SHAP are used for local and global interpretation, the review highlights both their practical promise and their methodological constraints, including computational overhead, dataset dependence, and deployment challenges in resource-constrained or real-time environments. Rather than claiming a fully validated new detection framework, the present study identifies recurring gaps in the literature and uses them to motivate a more focused future research agenda. Accordingly, the following directions are organized around improving interpretability efficiency, strengthening robustness under dataset shift, and enhancing the operational usability of XAI in cybersecurity defense. In this sense, the contribution of the review is not the introduction of a new benchmarked model, but the structured synthesis of current evidence and the clarification of high-priority directions for future XAI-driven malware-analysis research. To avoid an overly broad future-work discussion, the directions below are prioritized according to the main limitations exposed in the present review, namely computational overhead, limited generalization evidence, insufficient operational grounding, and the need for privacy-aware and robust XAI deployment. First, more work is needed on computationally efficient XAI for high-dimensional malware-analysis data. A practical hypothesis is that lightweight or approximate explanation strategies can preserve most of the interpretive value of SHAP and LIME while reducing latency and memory burden to a level more suitable for operational deployment. A preliminary evaluation design would compare explanation methods under standardized runtime, memory, and feature-space conditions across traffic-oriented benchmark datasets. Second, future research should examine generalization and explanation stability under dataset shift. The central hypothesis is that feature-attribution patterns derived from one benchmark environment may not remain stable across different device ecosystems, network conditions, attack compositions, or collection periods. A feasible preliminary design would involve cross-dataset comparison, temporal split validation, and consistency analysis of explanation outputs under distributional change. Third, XAI for malware analysis should be made more operationally actionable for analyst-facing decision support. The key hypothesis is that explanation outputs become more useful when they are explicitly linked to downstream actions such as alert prioritization, firewall or IDS rule adjustment, signature updating, and forensic escalation. A preliminary study design could evaluate workflow-oriented explanation interfaces in simulated SOC scenarios or analyst-in-the-loop review settings. Fourth, future work should further explore robust and privacy-aware XAI frameworks. This includes federated XAI settings, where interpretability must be preserved without centralizing sensitive data, as well as adversarial robust explanation methods that remain reliable under obfuscation, evasion, or poisoning attempts. A practical hypothesis is that combining privacy-preserving learning with explanation-stability constraints may improve both deployment feasibility and trustworthiness in security-sensitive environments.

1. Innovative research avenues that overcome the constraints of current techniques
  a. Distributed interpretability modeling within a federated learning framework.
    It is essential to create a collaborative optimization system that prioritizes privacy and model interpretability, along with a Nash equilibrium resolution framework grounded in game theory. The primary goal is to address the consistency issue between local explanations and the overarching model in federated learning contexts, investigating joint optimization strategies involving Shapley values and differential privacy, while also overcoming computational limitations (Saxena & Mittal, 2025; Feng et al., 2023). A complementary direction is the integration of Federated Learning (FL) with Explainable AI (XAI) for malware detection in privacy-sensitive environments. Shallom & Ikemefuna (2025) proposed a federated deep learning framework trained across decentralized clients using feature vectors from binaries and behavioral logs, while incorporating SHAP and LIME for interpretability. Their evaluation demonstrated that FL-XAI systems can achieve detection rates comparable to centralized approaches, but with the advantage of preserving data confidentiality and providing transparent explanations at both local and global levels. This architecture was shown to be particularly effective for domains such as healthcare and finance, where regulatory requirements restrict raw data sharing. The study highlights FL-XAI as a scalable, privacy-preserving solution capable of delivering trustworthy threat intelligence across heterogeneous environments (Shallom & Ikemefuna, 2025).
  b. Adaptive interpretability enhancement mechanism for dynamic environments
    Combine reinforcement learning and online learning principles to develop an incremental interpretability framework that continuously adapts. The main challenge is to enhance time series modeling capabilities, create a time series anomaly detection system that can dynamically track SHAP values, and provide real-time interpretability responses to emerging attack patterns (such as side-channel and time-based covert attacks) (Luo & Surdeanu, 2023).
  c. Development of a cross-domain interpretability evaluation system
    Create a multi-faceted evaluation framework that includes quantitative metrics and cognitive bias assessments. This involves designing an evaluation model that incorporates the cognitive traits of human experts, introduces robustness tests against adversarial samples, and establishes a three-dimensional evaluation system that encompasses various attack types, platform architectures, and data distributions (Bulgakova et al., 2025; National Institute of Standards and Technology, 2012).
2. Comprehensive research on broadening attack scenarios and platform coverage
  a. Document-type malware: Utilize NLP and structured parsing technologies to create context-aware hybrid interpretation methods (Quinn et al., 2022).
  b. Supply chain attacks: Develop a recursive interpretability mechanism to enable causal chain tracking through multi-layer code abstraction (Shree et al., 2024).
  c. Database attacks: Combine symbolic execution and query parsing techniques to build a logical path visualization tracking framework (Su & Wassermann, 2006; Guo et al., 2024).
  d. Research on adapting to heterogeneous platforms: Broaden cross-platform verification to include macOS/Linux kernel systems and IoT firmware and create a platform adaptation model that accounts for differences in instruction sets and system call characteristics. The emphasis is on examining the stability variations of interpretation tools across different architectures and developing kernel-level feature attribution techniques (Johnstone & Akinfaderin, 2025; Mahdavifar, Alhadidi & Ghorbani, 2022).
3. Integrated innovation of dynamic and situational awareness capabilities
  a. Real-time optimization of defense scenarios
    Establish a stress testing environment to assess the runtime efficiency of interpretation methods and create a lightweight interpretation framework tailored for edge computing. The focus is on enhancing real-time decision support capabilities amidst high-frequency attack traffic, achieving millisecond-level detection of abnormal paths and interpretation generation (Izuazu et al., 2025; Xydas et al., 2006; Mahdavifar et al., 2020b).
  b. Modeling complex attack scenarios
    Create a multimodal attack signal fusion engine to incorporate non-explicit timing data, such as execution time and power consumption characteristics. The key objective is to advance context-aware methods based on tensor decomposition and establish a dynamic mapping relationship between attack behavior patterns and interpretation outputs (Aizpurua, Palmer & Orús, 2025; Slack et al., 2020).
4. Construction of System-level Integration and Verification Framework
   A Deep integration with the new architecture
    Formally verify and integrate XAI technology with Software-defined Security (SDS) and Intent Networking (IBN). The focus is on solving the issue of architectural compatibility and developing an interpretation-driven decision-making module that supports the automatic generation of policies (E-SPIN, 2023).
  b. Research on Model Structure Adaptation
    The system compares the interpretability differences between traditional classifiers and deep models, and establishes a model structure-interpretation quality correlation model. Focus on analyzing the essential differences in interpretation stability between symbolic models and neural networks, and develop a unified interpretation paradigm for hybrid models (E-SPIN, 2023; Zakeri et al., 2025; Birahim et al., 2025).
5. Enhanced robustness in adversarial scenarios
  a. Attack sample defense mechanism
    Construct a joint optimization framework of adversarial training and interpretation optimization and develop an adaptive detection module of obfuscation technology. The focus is on studying the influence mechanism of attack sample perturbation on the interpretation results and designing a robust enhanced feature attribution algorithm (Pawlicki et al., 2024).
  b. Zero-day attack response strategy
    Explore unsupervised interpretation methods based on abnormal pattern mining and develop an interpretability inference framework for unknown attack types. The key is to break through the visualization technology of the implicit feature space and achieve the causal chain reconstruction of zero-day attacks (Devi et al., 2024).

This technology future direction of development, by establishing a four-dimensional development path of “breakthroughs in basic theories—scenario expansion—system integration—confrontation enhancement”, effectively promotes the gradual advancement of XAI technology from the laboratory verification stage to a practical defense system, thereby laying the foundation for intelligent defense with cognitive computing capabilities for cyberspace security. In the subsequent research work, emphasis should be placed on key areas such as cross-level technical collaboration, innovation of formal verification methods, and the construction of a standardized evaluation system (Rjoub et al., 2023; Kumar et al., 2025; Mohale & Obagbuwa, 2025).

In recent years, strengthening malware detection and network intrusion detection systems has become very important. Naeem propose an optimization-based graphical approach to improve malware detection performance on consumer devices through an ensemble learning model (Aldehim et al., 2023). At the same time, Ullah explored an intrusion detection method that combines big multimodal data with transfer learning, and optimized network security strategies in the IoT environment through game theory (Naeem et al., 2024). The same research team also developed the NIDS-VSB system, which utilizes Spark big data optimization and transfer learning to perform efficient intrusion detection for Connected Vehicle (VANET) scenarios. On the other hand, Zhao propose a hybrid approach based on transfer learning and generative adversarial samples (GANs), which further improves the accuracy and robustness of malware detection through graphical representation (Ullah et al., 2024a). These studies show that the combination of advanced machine learning technology and big data optimization methods can effectively address modern network security challenges and provide efficient and reliable protection schemes for complex network environments (Ullah et al., 2024b). Another recent advancement in explainable malware detection has focused on adaptability to unseen malware variants. Khan proposed a lightweight, interpretable Random Forest–based system trained on a single malware subtype (Transponder from the Spyware family) yet capable of detecting 15 unseen malware subtypes from the CIC-MalMem-2022 dataset. The system achieved over 99.8% accuracy with an average processing time of 5.7 µs per file, demonstrating both efficiency and scalability. Importantly, the framework relied on only the top five most influential features, improving interpretability while reducing computational overhead. The incorporation of SHAP further enhanced model transparency, allowing security analysts to understand decision-making processes. This work not only demonstrates the feasibility of generalizing malware detection to novel subtypes with minimal training data but also underscores the necessity of integrating accuracy, efficiency, and interpretability into future malware detection frameworks (Zhao et al., 2025).

The figure shows the research direction and content of malware in the field of detection, including six main contents, as outlined in Table 4.

- Android malware detection (such as deep learning, defense mechanisms, and classification interpretation)
- AI and machine learning methods (such as hybrid models, Shapley value analysis, and integrated algorithms)
- Interpretable AI and visualization (such as transparency interpretation, attention mechanisms, and cloud computing)
- Windows malware detection (such as PCA-LSTM techniques and call graph analysis)
- Cross-platform and hybrid technologies (such as desktop and mobile device detection, graph representation learning)
- Behavior and context analysis (such as concept drift forensics and APT author verification)

**Table 4:** Survey and review articles on malware analysis and XAI and technical research articles on malware detection approaches.

Article	Problem	Solution	Shortcoming	Year
(A)				
A Survey of Android Malware Detection with Deep Neural Models (Mohale & Obagbuwa, 2025)	How to use deep neural networks to detect and classify Android malware	Provides a review of different deep neural network architectures (*e.g*., FCN, CNN, RNN), highlights advances in DL for Android malware detection, and discusses future research	DL models may be less effective and computationally complex when data is scarce or of low quality	2021
Deep Learning for Android Malware Defenses: A Systematic Literature Review (Qiu et al., 2021)	How to use deep learning to defend against Android malware	Systematically reviewed DL-based detection and defense methods, covering architectures and feature processing techniques, highlighting trends like reinforcement/federated learning	DL models may lack transparency and robustness, and have high computational complexity	2022
Malware Detection with Artificial Intelligence: A Systematic Literature Review (Liu et al., 2022b)	How to detect malware using AI	Systematic review of AI methods applied to malware detection	Limited in platform-specific details, little focus on interpretability	2024
A Survey of Recent Advances in DL Models for Detecting Malware in Desktop and Mobile Platforms (Liu et al., 2022a)	How to improve malware detection on desktop and mobile platforms	Surveyed DL methods across Windows, Linux, and Android	Limited by platform-specific challenges and DL transparency	2024
A review of deep learning models to detect malware in Android applications (Alfs et al., 2019)	How to improve Android malware detection using ensemble DL + XAI	Comprehensive review based on CIC-AndMal2020 dataset, integrated DL + XAI	High complexity may limit real-time applications on large datasets	2023
A Survey and Evaluation of Android Malware Evasion Techniques and Detection Frameworks (Tansen & Nurdiarto, 2020)	How Android malware evades detection systems	Surveys evasion techniques and evaluates detection frameworks	Limited focus on proposing new countermeasures	2023
Explainable artificial intelligence for cybersecurity: a literature survey (Smmarwar, Gupta & Kumar, 2023)	Limited understanding of AI decision-making in cybersecurity	Surveys methods and applications of XAI for security	More theoretical, lacks implementation details	2022
Explainable AI Applications in Cyber Security: State-of-the-Art in Research (Alalayah et al., 2023)	Insufficient use of XAI in practice	Reviews state-of-the-art XAI applications in cybersecurity	Limited discussion on overcoming technical barriers	2022
Explainable AI for Android Malware Detection: Towards Understanding Why the Models Perform So Well (Jorgensen et al., 2024)	Difficulty in interpreting why AI models perform well in malware detection	Uses XAI techniques to analyze decision-making	Focused on explaining model performance rather than improving detection	2022
Machine Learning in Malware Analysis: Current Trends and Future Directions (Zhang et al., 2024)	Rapid malware evolution challenges ML effectiveness	Reviews ML trends and future directions in malware detection	More focused on theory than actionable frameworks	2024
Comprehensive Review on Cybersecurity: Modern Threats and Advanced Defense Strategies (Huang et al., 2021)	Modern cyber threats outpace traditional defenses	Reviews advanced defense strategies	Limited depth on emerging threats such as quantum computing	2024
A Critical Review of Emerging Cybersecurity Threats in Financial Technologies (Wang, He & Zhang, 2018)	FinTech faces unique evolving threats	Critically reviews threats and tailored defenses	Limited applicability beyond financial sector	2024
A Systematic Literature Review of Android Malware Detection Using Static Analysis (Sasidharan & Thomas, 2021)	Static analysis struggles with obfuscation	Systematically reviews Android static analysis methods	Little focus on hybrid/dynamic methods	2020
Static analysis of malware in Android-based platforms: A progress study (Jeng et al., 2022)	Growing complexity and volume of Android malware	Reviews progress in Android static analysis	Static analysis struggles with obfuscation/encryption	2021
Android Platform Malware Analysis (Zhang et al., 2024)	Lack of comprehensive understanding of Android malware trends	Reviews Android malware samples and attack techniques	Limited adaptability to novel/emerging malware	2015
(B)				
Identifying Android Malware Using Network-Based Approaches (Kumar et al., 2025)	Distinguishing malicious *vs* benign apps with limited data	Proposed weighted network + label propagation	Depends heavily on label propagation, accuracy drops with insufficient data	2019
Why an Android App Is Classified as Malware (Gupta & Seeja, 2024)	How to interpret malware classification	Introduced XMal with attention mechanism for interpretability	Some misclassifications remain, relies on feature engineering	2021
Explainable Machine Learning for Malware Detection Using Ensemble Bagging Algorithms (Gaber, Ahmed & Janicke, 2024)	Improving accuracy and interpretability	Used ensemble bagging + SHAP, achieving 98.1% accuracy for zero-day malware	Requires extensive feature engineering	2022
Hybrid Machine Learning Meta-Model Based on Contextual and Behavioral Representations (Maniriho, Mahmood & Chowdhury, 2024)	Improve Windows PE malware detection	Hybrid ML model combining context, static, behavioral features	Computationally intensive	2022
Decoding the Secrets of ML in Windows Malware Classification (Mbunge et al., 2023)	How dataset/feature choice affects detection	Found static features outperform dynamic; balanced samples improved generalization	Declines as malware family diversity increases	2023
Memory Visualization-Based Malware Detection Technique (Faruki et al., 2023)	Difficulty detecting obfuscated/memory-resident malware	Converted memory dumps to visual patterns	Resource-intensive, slow for real-time	2022
A Novel DL-Based Attention Mechanism for Android Malware Detection and Explanation (Charmet et al., 2022)	Improve CNN accuracy and explainability	Applied attention + improved LIME for opcode images	Limited to opcode image visualization	2022
A Robust CNN for Malware Classification against Executable Adversarial Attack (Zhang et al., 2022a; Ali & Abdul-Qawy, 2021)	Accurate PE malware classification using function call graphs	Developed MalSensor (98.35% accuracy, 0.7 s classification)	Reliant on static analysis	2020/2024
Drift Forensics of Malware Classifiers (Altaha & Riad, 2024)	Analyzing concept drift in malware detection	Forensic framework to identify POI in classifier drift	Long-term effectiveness uncertain	2023
Enhancing State-of-the-Art Classifiers (FINER framework) (Obi et al., 2024; Umoga et al., 2024)	Improving interpretability and transparency of DL classifiers	Proposed FINER framework with feature attribution + multitask learning	Complex, requires expert knowledge	2020/2023
A Journey Through Android App Analysis (Pan et al., 2020)	Understanding app vulnerabilities	Developed analysis tools (Dexpler, FlowDroid, AndroZoo)	Static analysis may ignore dynamic behavior	2021
A framework for Android Malware Detection and Classification (Alfalqi, Alghamdi & Waqdan, 2015)	Analyze malware in network traffic	Combined Random Forest + SHAP on Drebin/Genome datasets	Dependent on traffic quality	2018
PAIRED: An Explainable Lightweight Android Malware Detection System (Wu et al., 2021)	Lightweight high-accuracy Android malware detection	Used SHAP + minimal features, 98% accuracy	Limited to static indicators	2022
XAI-AMD-DL: Explainable AI for Android Malware Detection (Kumar & Subbiah, 2022)	Use XAI + DL for malware detection	Hybrid CNN-BiGRU + SHAP, accuracy 97.98%	Requires high computational resources	2023
Learning-Based Artificial Algae Algorithm for Malware Detection (Trizna, 2022)	Improve interpretability of HPC-based intrusion detection	Proposed HPCDR framework	Needs dedicated hardware	2023
Extensible ML for Encrypted Network Traffic Labeling (Dambra et al., 2023)	Classify VPN-encrypted traffic	ML framework with uncertainty quantification	Limited by VPN protocol variety	2022
OSINT for Malicious Behavior Discovery and Interpretation (MAMBA) (Shah, Jamil & Khan, 2022)	How to use OSINT for malicious behavior	Linked API calls to ATT&CK tactics using DL + interpretability	Dependent on ATT&CK database integrity	2022
Demadroid: Object Reference Graph-Based Detection (Jorgensen et al., 2024)	Detect complex code structures in Android apps	Used object reference graphs	Computationally heavy, sensitive to variation	2018
ProDroid: HMM-based Android Malware Detection (Chow et al., 2023)	Detect evolving malware behaviors	Used profile Hidden Markov Models	Relies heavily on training data	2021

Pham introduced a multimodal Windows malware detection framework that integrates hybrid static and dynamic analysis with enriched graph representations. The approach leverages CNNs for static PE features and GNNs for dynamic API call sequences, enriched by BERT-based embeddings for semantic context. Importantly, the framework incorporates XAI methods (SHAP, LIME, PGExplainer) to interpret classification outcomes, providing both global and local explanations of malicious behaviors. Their results demonstrated near-perfect accuracy (99.9%) while significantly improving transparency, positioning it among recent representative explainable solutions for Windows malware detection (Madamidola, Ngobigha & Ez-zizi, 2025).

More recently, Zhang introduced an innovative hybrid framework that integrates Transfer Learning (TL) with Explainable AI (XAI) for malware detection (Zhang et al. 2022a). By leveraging pre-trained neural networks, the system effectively adapts to emerging malware with minimal training data, addressing the challenge of data scarcity in evolving threat landscapes. The integration of XAI methods further ensures transparency and trustworthiness, enabling security analysts to understand and validate the model’s predictions. Experimental evaluations across multiple datasets confirmed that this approach not only improves detection accuracy but also provides interpretable insights into decision-making processes. This work represents a significant step forward in balancing performance and interpretability, reinforcing the role of hybrid TL–XAI strategies in combating dynamic cyber threats (Pham et al., 2025).

Most of these research directions and content use new technologies to pinpoint malware detection while improving cross-platform capabilities. Recent research has also expanded the scope of malware detection frameworks by integrating federated learning with XAI. For instance, Ciaramella et al. (2025a) proposed a Windows-focused detection system that leverages federated machine learning (FML) and integrates Grad-CAM to visualize influential features, thereby enhancing explainability and complying with privacy regulations. Similarly, Dominic & Ranjan (2025) introduced a privacy-preserving malware detection framework that combines federated learning with SHAP and LIME for explainability, achieving high accuracy in a decentralized environment while safeguarding confidentiality (Subbarao, Togaru & Yogi, 2025; Ciaramella et al., 2025a).

Table 5 provides a comparative summary of existing survey and review studies on malware detection. Prior works are often restricted in scope: some focus exclusively on Android and deep learning methods while others adopt a broader AI-based perspective but devote little attention to interpretability. More recent surveys have extended the discussion to adversarial robustness or hybrid learning strategies, whereas others concentrate on specific domains such as IoT malware or malicious Microsoft Office documents. Only a limited number of studies explicitly incorporate explainable AI, either within Android malware detection or through graph-based approaches.

**Table 5:** Comparative summary of survey and review articles.

Reference	Windows	Android	XAI Focus	Coverage breadth
*A Survey of Android Malware Detection with Deep Neural Models* (Qiu et al., 2021)	–	✓	–	Narrow (Android only)
*Deep Learning for Android Malware Defenses* (Gaber, Ahmed & Janicke, 2024)	–	✓	–	Broad on Android defenses
*Malware Detection with Artificial Intelligence: A Systematic Literature Review* (Maniriho, Mahmood & Chowdhury, 2024)	✓	✓	–	Broad AI focus, limited on interpretability
*Deep Learning Models for Detecting Malware in Desktop and Mobile Platforms* (Obi et al., 2024)	✓	✓	–	Cross-platform, no explicit XAI focus
*Survey on Explainable AI for Cybersecurity* (Devi et al., 2024)	✓	–	✓	Broad cybersecurity context, not platform-specific
*A Survey of Malware Detection Using Deep Learning Techniques* (Baghirov, 2025)	✓	✓	–	Comprehensive DL survey, no focus on XAI
*Explainable AI in Mobile Malware Detection: A Systematic Review* (Manthena et al., 2025)	–	✓	✓	Android-focused with strong emphasis on XAI
*Malware Threats in IoT and Mobile Ecosystems: A Review* (Alvi & Jalil, 2025)	Partial (IoT devices on Windows base)	✓	–	IoT + Android ecosystem, not XAI specific
*Adversarial Attacks and Defenses in Malware Detection: A Survey* (Dasila et al., 2025)	✓	✓	–	Emphasis on adversarial robustness
*Hybrid Learning Approaches for Malware Analysis: A Survey* (Hartman & Rimal, 2025)	*✓*	*✓*	*–*	*Hybrid ML/DL overview, little on interpretability*
*Recent Advances in Malware Detection: Graph Learning and Explainability* (Shokouhinejad et al., 2025)	–	*✓*	*✓*	*Graph learning + XAI, both platforms*
*Interpretable ML for Mitigating Feature-Driven Attacks* (Oyedotun, Oise & Ozobialu, 2025)	*✓*	*–*	*✓*	*Narrow (malicious documents, macros)*
*This Study Transforming Malware Analysis with Explainable AI: A Detailed Review of Windows and Android Platforms*	*✓*	*✓*	*✓*	*Dual-platform + dedicated XAI analysis (LIME/SHAP) with dataset evaluation*

In contrast, the present study offers a dual-platform analysis of Windows and Android malware with a dedicated emphasis on explainable AI techniques (LIME and SHAP). By combining cross-platform coverage with interpretability and validating the findings through benchmark experiments, this study delivers a more comprehensive synthesis than prior surveys and establishes its distinct contribution to the field.

Wazid proposed a secure deep learning-enabled malware detection system for IoT-based intelligent transportation systems (ITS), known as SDLMA-IITS. By combining multiple deep learning models with SHAP-based explanations, the framework achieved over 99% accuracy and demonstrated resilience against various attack scenarios. Their work highlights how explainable AI enhances feature attribution and trustworthiness in critical infrastructure contexts, particularly within ITS environments where both reliability and transparency are indispensable (Dominic & Ranjan, 2025).

Building on this line of research, Wazid developed an explainable deep learning-enabled malware attack detection framework for IoT-enabled ITS, further integrating XAI into model evaluation. Their system not only provided robust classification accuracy but also delivered interpretable insights into influential traffic features, facilitating security analysis and practical deployment in intelligent transportation environments. This underscores the applicability of XAI in domains requiring both real-time response and model accountability (Ciaramella et al., 2025b).

Beyond desktop and mobile environments, researchers have investigated domain-specific deployments. Alohali developed a two-stage malware detection model for Internet of Vehicles (IoV), combining BiLSTM-MHSA classifiers with SHAP-based explanations to enhance transparency and accuracy in vehicular networks. This demonstrates the versatility of XAI approaches in emerging intelligent transportation ecosystems, where both privacy and interpretability are critical (Wazid et al., 2025).

In the context of IoT security, Sharma proposed a deep learning-based ensemble intrusion detection framework (DLEX-IMD) to address malware attacks in IoT-enabled environments. The model was trained and evaluated on benchmark datasets, achieving 99.96% accuracy and an F1-score of 0.999. To enhance trust and interpretability, the framework incorporated LIME explanations, which justified the reliability of its training process by highlighting influential features in classification outcomes. Compared with several existing schemes, DLEX-IMD consistently outperformed alternatives, thereby illustrating the effectiveness of combining deep ensemble learning with XAI techniques for IoT malware detection (Alohali et al., 2025).

Towards the later stage of related work, Bagirov conducted a comprehensive investigation into robust malware detection using XAI, focusing on Android malware detection with interpretable machine learning models. Using the CICMalDroid dataset, the study applied Logistic Regression and multiple tree-based algorithms, achieving an overall F1-score of 94%. More importantly, the research emphasized that XAI does not merely serve to improve predictive accuracy but also provides valuable interpretability by identifying critical features influencing classification outcomes. This contribution demonstrates that enhancing model transparency and reliability is essential for practical deployment, further reinforcing the need for explainable approaches in malware analysis beyond pure performance metrics (Mittal et al., 2025).

## Conclusion

This article systematically investigates recent malware analysis and detection technologies for Windows and Android platforms, with a focused emphasis on the role of XAI in enhancing modern detection frameworks. By combining a structured review of state-of-the-art methods with experimental observations on multiple publicly available datasets, this study derives concrete conclusions regarding the effectiveness, limitations, and practical value of XAI-assisted malware detection.

The main findings of this work confirm that while conventional network-based and behavioral-feature-driven detection approaches remain effective for identifying malicious activities, they exhibit inherent limitations in scalability, adaptability to evolving malware variants, and decision transparency. Our analysis demonstrates that deep-learning-based models achieve strong detection performance but function largely as black boxes, which restricts their practical usability in operational cybersecurity environments. In contrast, the integration of XAI techniques—specifically LIME and SHAP—enables clear attribution of model decisions to high-impact behavioral and traffic features, thereby transforming opaque detection outcomes into interpretable and actionable insights.

A key result of this study is that XAI-based interpretation can effectively uncover the underlying behavioral logic of malware attacks, distinguish dominant malicious characteristics from redundant features, and support the validation of detection rules across heterogeneous attack scenarios. The experimental observations further indicate that explainability mechanisms significantly enhance analyst trust and situational awareness, bridging the gap between high detection accuracy and real-world deployment requirements. These findings highlight that interpretability is not merely an auxiliary property, but a functional component that directly improves the reliability and usability of malware detection systems.

Nevertheless, this work also identifies critical unresolved challenges. The rapid emergence of polymorphic malware, adversarial crafted samples, and zero-day threats continues to undermine the generalization capability of existing models, including XAI-enhanced frameworks. Moreover, the increasing prevalence of IoT, cloud-based, and mobile infrastructures introduces highly heterogeneous traffic patterns, placing additional computational and scalability constraints on both detection and explanation modules.

In conclusion, this study establishes that the deep integration of explainable artificial intelligence with traditional and deep-learning-based malware detection techniques constitutes a necessary and promising direction for next-generation cyber-defense systems. By simultaneously improving detection effectiveness and decision interpretability, XAI-driven frameworks provide a more transparent, trustworthy, and operationally viable foundation for malware defense across personal devices, enterprise networks, and critical infrastructure. Future research should prioritize computationally efficient explainability, broader real-world data coverage, privacy-preserving collaborative learning, and adversarial robustness to further advance the development of scalable and trustworthy malware detection systems. The findings synthesized in this review should be interpreted as literature-based observations rather than as fully validated claims of causal performance improvement, broad external generalizability, or formally benchmarked interpretability robustness.

## Supplemental Information

10.7717/peerj-cs.3902/supp-1 Supplemental Information 1 DDoS ICMP Flood.

10.7717/peerj-cs.3902/supp-2 Supplemental Information 2 DDoS UDP Flood.

10.7717/peerj-cs.3902/supp-3 Supplemental Information 3 DoS ICMP Flood.

10.7717/peerj-cs.3902/supp-4 Supplemental Information 4 DoS UDP Flood.

10.7717/peerj-cs.3902/supp-5 Supplemental Information 5 MITM ARP Spoofing.

## Additional Information and Declarations

### Data Availability

The following information was supplied regarding data availability:

The raw data analyzed in this study is available at the Canadian Institute for Cybersecurity at the University of New Brunswick: https://www.unb.ca/cic/datasets/iomt-dataset-2024.html.
